# A home-based exercise programme attenuates fatigue in primary biliary cholangitis: Results from the EXCITED clinical trial

**DOI:** 10.1016/j.jhepr.2024.101210

**Published:** 2024-09-06

**Authors:** Alice Freer, Felicity R. Williams, Simon Durman, Jennifer Hayden, Matthew J. Armstrong, Palak J. Trivedi

**Affiliations:** 1 National Institute of Health Research (NIHR) Birmingham Biomedical Research Centre (BRC), Centre for Liver and Gastrointestinal Research, University of Birmingham, Birmingham, UK; 2 Liver Unit, University Hospitals Birmingham Queen Elizabeth. Birmingham UK; 3 School of Sport, Exercise and Rehabilitation Sciences, University of Birmingham, Birmingham, UK; 4 Institute of Immunology and Immunotherapy, University of Birmingham, Birmingham, UK; 5 Institute of Applied Health Research, University of Birmingham, UK

**Keywords:** Autoimmune Liver Disease, Cholestasis, Functional Capacity, Health Related Quality of Life, Patient Reported Outcome Measure, Symptoms

## Abstract

**Background & Aims:**

Fatigue is a commonly reported symptom of primary biliary cholangitis (PBC). We conducted a single-arm, open-label clinical trial to assess the efficacy of a physiotherapist-led home-based exercise programme (HBEP) in patients with PBC and moderate-to-severe fatigue (NCT04265235).

**Methods:**

A 12-week individualised HBEP (aerobic + resistance based) was delivered to patients with a PBC-40 fatigue domain score ≥33. The primary efficacy outcome measure was a reduction in fatigue severity by ≥5 points. Secondary outcome measures included other domains of PBC-40, the FIS (fatigue impact scale), ESS (Epworth sleepiness score), HADS (hospital anxiety and depression scale), aerobic capacity (ISWT [incremental shuttle walk test], Duke activity status index (predicted VO₂ peak) and physical function (short physical performance battery [SPPB]).

**Results:**

A total of 31 patients were recruited, of whom 30 completed the 12-week HBEP (29 women; median age 53 years, median alkaline phosphatase value: 1.5x the upper limit of normal, median bilirubin: 12 μmol/L, and median baseline PBC-40 fatigue score 42). The primary outcome was met by 26 patients, with a median reduction in PBC-40 fatigue score of -10.5 points (IQR -9 to -13; *p <*0.001). Reductions were also observed in the symptom, cognition, and emotion domains of PBC-40, and in the FIS, ESS and HADS (*p <*0.01 for all measures). This was alongside increases in the median ISWT (+90 m; IQR 57.5-110), predicted VO₂ peak (+2.41 ml/kg/min; IQR 0.01-4.05), and SPPB (+1 point; IQR 0-1.4) (all *p <*0.001). 28 participants achieved the maximum SPPB score of 12/12 (*vs.* 13 patients at baseline; *p <*0.001). No significant adverse events were reported.

**Conclusion:**

This proof-of-concept study shows that a HBEP is safe, feasible, and has the potential to attenuate fatigue. Controlled trials are needed to validate the efficacy of exercise interventions in PBC.

**Impact and implications:**

Fatigue is a common symptom in primary biliary cholangitis (PBC), and is linked to cognitive dysfunction, somnolence, and reduced activity. The pathogenesis is multifactorial, and muscle bioenergetic abnormalities have been proposed to contribute. In this study, we show that a home-based exercise programme, consisting of aerobic and resistance-based sets, can be safely delivered to people living with PBC. In addition, the programme led to a reduction in fatigue severity, less daytime sleepiness and improved cognitive function.

## Highlights

• Fatigue is a commonly reported symptom of primary biliary cholangitis.
• Presently, there are no effective medical treatments for fatigue in primary biliary cholangitis.
• We show that a 12-week home-based exercise programme was associated with significant reductions in fatigue severity.
• Larger confirmatory trials of exercise interventions in chronic cholestatic diseases are warranted.

## Graphical abstract

**Figure:**
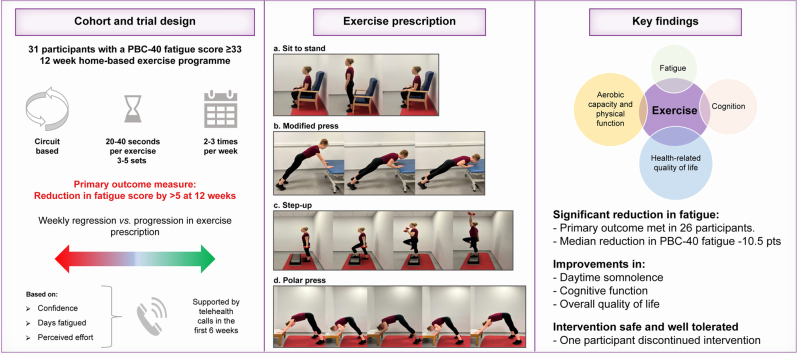

## Introduction

Primary biliary cholangitis (PBC) is a rare chronic cholestatic disease, in which clinical outcomes are dictated by progression to cirrhosis and need for transplantation.^1^^,^^2^ Effective bile acid therapy is associated with improved transplant-free survival and lower rates of liver cancer, but with limited impact on symptom burden.[3], [4], [5], [6], [7], [8] Whilst considered a rare disease, the impact on patient quality of life is substantial, with symptoms dominated by pruritus, cognitive impairment, and fatigue.[9], [10], [11], [12] The latter can be severe and debilitating, and is reported by 40-80% of patients across the UK-PBC population.^3^ Whilst fatigue is not an indicator of liver disease severity, it is associated with heightened mortality risk.^13^^,^^14^ Moreover, there are no effective pharmacological interventions for fatigue in PBC, and >40% of patients have persistent and significant symptoms following liver transplantation.[15], [16], [17]

Fatigue in PBC is not thought to be the result of a single activity or challenging functional task, but rather an inability to perform sustained activities or movements over time. The cause of fatigue in cholestasis is likely to be multifactorial, with contributions from both central (*i.e.* decrease in voluntary activation of muscle relating to a decline in motor cortex drive) and peripheral factors (*i.e.* decrease in contractile strength and endurance relating to altered muscle action potential mechanisms).[18], [19], [20], [21], [22], [23], [24], [25] Peripherally, muscle bioenergetic abnormalities, alongside suppressed anaerobic threshold during physical exertion, have been proposed.^22^^,^^23^ For example, when compared to healthy controls, patients with PBC-associated fatigue manifest delayed peripheral muscle pH recovery and excessive acidosis following ≤35% maximal voluntary contraction of lower leg muscles.^22^ Despite this, mechanistic studies show that repeated exercise can attenuate peripheral discrepancies, which may improve exercise tolerance.^22^

Exercise intervention has been shown to attenuate central fatigue in healthy individuals and those with long-term neurological, respiratory, and oncological conditions.^23^^,^[26], [27], [28], [29], [30], [31], [32] This may also be relevant to individuals with PBC, who concomitantly report memory and cognitive decline, increased daytime somnolence and low mood, attributed to abnormalities in central activation of cortical inhibitory and excitatory circuits, resulting in central nervous system dysfunction.^33^ The use of repetitive/progressive exercise programmes at a moderate intensity (safe zone training) allows for steady adaptation at a muscular level, and improvement in exercise tolerance through physiological acclimation.^34^ This, combined with the hypothesis that repeated exercise may attenuate altered muscle bioenergetics, or improve central processes, offers an opportunity to explore the impact of a structured, moderately intense exercise programme on fatigue in cholestasis.

The overarching goal of the study was to conduct an open-label clinical trial to evaluate the impact of a physiotherapy-led exercise initiative, specifically to attenuate fatigue associated with PBC. Importantly, this was a home-based exercise programme (HBEP), designed specifically to overcome logistical, travel and cost challenges associated with a hospital-based setting.

## Patients and methods

A single-arm (open-label) trial was designed to investigate the impact and safety of a 12-week HBEP; specifically on fatigue, health-related quality of life (HRQoL), sleep quality, aerobic exercise capacity and physical function, in patients with PBC. Ethical approval was obtained from the National Research Ethics Service Committee in London and the Health Research Authority (Research Ethics Service reference number 18/LO/2109) and was approved by the Queen Elizabeth University Hospital Birmingham Research and Development Department. The study was registered with clinicaltrials.gov (NC04265235).

### Participant identification and eligibility

Detailed descriptors of the full study protocol and trial schema, including specific inclusion/exclusion criteria, the natur.e of patient and public involvement and the specific analysis plan are presented elsewhere (Supplementary File 1 ^35^ and Fig. S1). Briefly, as part of the Birmingham symptom-focussed specialist nurse programme, all individuals who attend PBC outpatient clinics complete a PBC-40 quality-of-life questionnaire as part of routine standard of care during outpatient clinic visits. This allowed timely identification and pre-screening of eligible participants with moderate-to-severe fatigue, as evidenced by a score of ≥33 in the fatigue domain of PBC-40 (a validated health-related quality-of-life tool for patients with PBC).^15^^,^^35^^,^^36^

Prior to starting any intervention, patients attended a dedicated screening visit (2 weeks after pre-screening indicated trial eligibility). This was to ensure stability in symptom severity, as indicated by persistently elevated PBC-40 fatigue scores ≥33 between visits. Individuals with additional contributors to fatigue were excluded; encompassing (but not limited to) those with anaemia (any cause), renal dysfunction, concomitant malignancy, untreated or poorly treated thyroid disease, other untreated or poorly treated extrahepatic autoimmune diseases, vitamin B or D deficiency, gluten-sensitive enteropathy, and untreated or poorly treated diabetes mellitus. Other key exclusion criteria were decompensated cirrhosis +/- portal hypertension, serum bilirubin values >50 μmol/L (in the absence of Gilberts syndrome), prior solid organ transplantation, a diagnosis of autonomic dysfunction, and/or cardiovascular instability.

### Intervention

Informed written consent was obtained from all participants prior to study entry. All participants underwent a baseline (visit 1) evaluation of aerobic exercise capacity/functional capacity (incremental shuttle walking test [ISWT], Duke activity status index [DASI]) and physical function (short physical performance battery [SPPB]) by a specialist liver physiotherapist.[37], [38], [39] These outcomes, alongside the participant’s confidence to complete the exercise guided the design of their individualised resistance programme; specifically, their entry level and number of sets (Table 1). To ensure a moderate intensity work rate was achieved, participants were educated on the Borg rate of perceived exertion (RPE) and provided a hand out with clear colour-coded training zones (white a prompt to increase intensity, green being optimal (RPE 12-14), amber a prompt to reduce intensity, and red to highlight their work effort was too high) (Fig. S2).^40^

**Table 1:** Levels of exercise with suggested work rate, rest phases and number of sets.

Level	Exercises	Work phase	Rest phase	Number of sets per exercise
1	Sit to stand	20 s	40 s	3-5
	Bench press			
	Frog squat			
	Bear crawl			
	Step-up			
2	Sit to stand	30 s	30 s	3-5
	Bench press			
	Frog squat			
	Bear crawl			
	Step-up			
3	Sit to stand	30 s	30 s	4-5
	Frog squat			
	Bear crawl			
	Kick sit			
	Body drop			
4	Frog squat	30 s	30 s	4-5
	Bear crawl			
	Kick sit			
	Body drop			
	Cobra			
	Polar press			
5	Frog squat	40 s	20 s	5
	Bear crawl			
	Kick sit			
	Body drops			
	Cobra			
	Polar press			

Prior to leaving the baseline visit, each participant completed their exercise programme under the supervision of the specialist, hospital-based liver physiotherapist. The latter is defined as an experienced clinician with a Bachelor of Science degree in Physiotherapy, with the ability to assess, treat, manage and prescribe advice on exercise prescription, fatigue management and lifestyle advice for patients with complex liver disease, ensuring the desired exercise intensity is possible to achieve. Importantly, it was essential that the physiotherapist had at least 12 months’ experience of working in a tertiary liver unit. Participants were then provided with a diary to record adherence/compliance and asked to complete their HBEP 2-3 times per week for 12 weeks. Importantly, the HBEPs were designed to be low impact and in a circuit format, without need for specialist equipment.

**Fig. 1:**
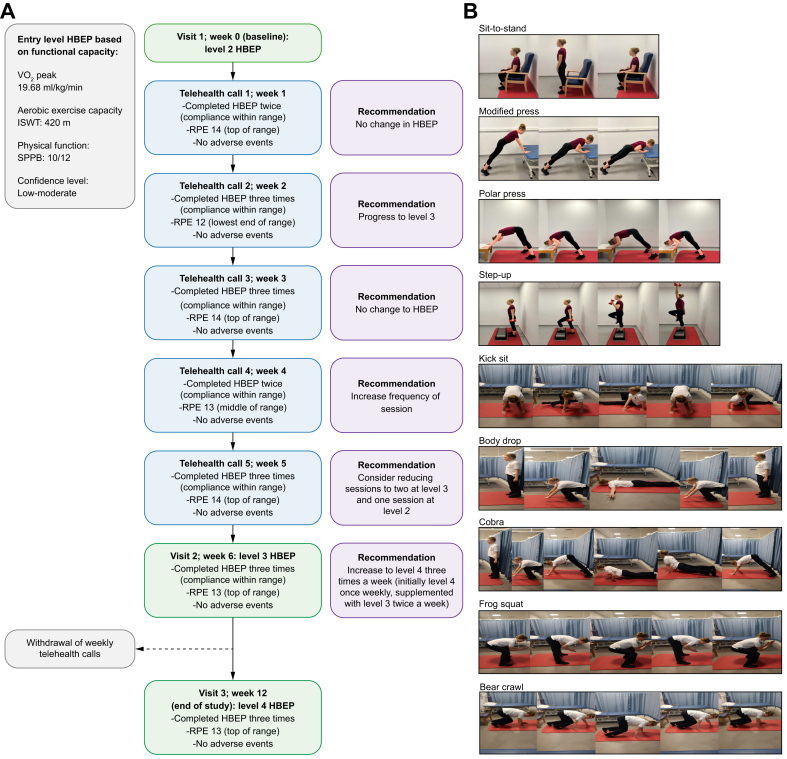
Representative exercise prescription. (A) An example of the initial exercise prescription based on functional capacity, aerobic exercise capacity, physical function and participant confidence level, progression/regression of programme based on compliance and work rate, following weekly telehealth calls between visits 1 and 2, and alterations of programme at visit 2 prior to routine scheduled telehealth calls being removed between visits 2 and 3. (B) Is indicative of resistance exercises that comprise the exercise prescription. HBEP, home-based exercise programme; ISWT, incremental shuttle walk test; RPE, rate of perceived exertion; SPPB, short performance physical battery.

Between weeks 1-5 (inclusive) of intervention, the specialist liver physiotherapist provided weekly structured ‘virtual’ telehealth consultations to each participant. These consultations evaluated participant engagement in the HBEP, highlighted limiting factors (such as mood) which may be influencing adherence, and whether any new symptoms of concern such as syncope, muscular pain or chest pain occurred. Following this discussion, the HBEP was reviewed with the participant. Dependent on responses to (a) confidence completing the programme, (b) ability to elicit a work rate of 12-14 using the RPE, (c) frequency of self-reported days with fatigue, the programme was progressed following each telehealth call (*i.e.*, reducing rest time between work phases/sets) or regressed (*i.e.*, increasing rest time between work phases/sets) between levels (Fig. 1). This was guided by patient fatigue to ensure that individuals were not overwhelmed by the HBEP.

Participants next attended the trial site at week 6 (visit 2) for clinical review and physical examination, followed by a further 6 weeks of HBEP (weeks 7 to 11) wherein the routine weekly telehealth consultations were withdrawn. A final study visit with clinical review was conducted at week 12 (visit 3). Participants were not limited from making other changes to their lifestyle (including dietary requirements) and were given standard healthy dietary advice throughout. Patients were advised to cease the programme if any adverse effects developed, such as chest discomfort, refractory itch, or syncope, and contact the trial team immediately.

### Outcome measures

The primary outcome measure was the proportion of patients attaining a reduction in PBC-40 fatigue domain score of ≥5 points at 12 weeks.^15^^,^^35^^,^^41^ The PBC-40 fatigue domain is widely accepted as an endpoint in HRQoL clinical trials, although there is lack of consensus as to what constitutes a minimum important change. Thus, our primary outcome measure was largely informed by prior interventional studies seeking to improve fatigue in PBC.^15^^,^^41^ Primarily, ours was an open-label pilot study, with the overarching goal of seeing if a HBEP was feasible, deliverable, and associated with a reduction in fatigue severity over a 12-week period. As such, the sample size was chosen pragmatically, based on published UK-PBC data, and powered to detect a mean change in the PBC-40 fatigue domain score of 5 units at 12 weeks (a difference associated with significantly greater levels of social function).^11^ Previous studies have shown a standard deviation of 8 units and a correlation of 0.6 between baseline and follow-up. Thus, using a power of 90% and significance level of 5% would require outcome data from 35 participants for an interventional arm in a 1:1 randomised-controlled trial, including a 10% attrition rate (if power was lowered to 80% because PBC is a rare disease, this would be 29 patients per arm). Thus, our intended sample size was a total of 40 patients for a single-arm study.

Key secondary outcome measures included the median change from baseline in the DFIS (daily fatigue impact scale),^42^ other domains of PBC-40 (emotional, social, and cognitive function, general symptoms and itch),^36^ the Epworth sleepiness score (ESS),^43^ the hospital anxiety and depression scale (HADS),^44^ impact fatigue had on cognitive function, score changes in the chronic liver disease questionnaire,^45^ and measures of aerobic exercise/functional capacity (incremental *s*huttle walking test [ISWT], Duke activity status index [DASI]) and physical function (SPPB [short physical performance battery]). All outcome measures were recorded at baseline, week 6 and week 12.

### Data analysis

Baseline characteristics were analysed using descriptive statistics and expressed as median (IQR), and n (%) for categorical data. Fisher’s exact test was used to analyse differences between categorical variables, and the Mann-Whitney *U* test for analysing differences between two groups of unpaired continuous variables. Wilcoxon signed ranked test was used to evaluate changes in continuous variables from baseline to week 6 and 12. Bonferroni-Dunn *post hoc* correction analysis was performed when performing multiple test evaluations on a single set of data. Logistic regression analysis was performed to identify factors associated with a ≥5 point reduction in the PBC-40 fatigue domain. Following completion of a Shapiro-Wilk’s test (*p* >0.05), a Pearson’s product-moment correlation was also used to assess the relationship between baseline, and post-intervention measures at weeks 6 and 12.

## Results

### Pre-screening and characteristics of the studied cohort

The study was conducted between April 2019 to March 2020. Of 261 pre-identified patients, 148 recorded a PBC-40 fatigue score ≥33, of whom 78 met study eligibility criteria. Thirty-four patients passed screening and agreed to take part, but due to emergence of the first wave of the COVID-19 pandemic, trial entry ceased after the 31^st^ consecutive participant was recruited. One participant withdrew from the study prior to week 6 due to unforeseen personal circumstances (Fig. S3). No significant differences were observed between trial eligible participants and those who were deemed eligible on pre-screening but who declined to take part in the study (Table S1).

Of the 30/31 participants who completed the 12-week HBEP, 97% were women, with a median age of 52 years (IQR 44-60 years) and PBC-40 fatigue domain score of 41.5 (IQR 37-43) (Table 2). Twenty-two participants were classified as having at least moderate cognitive symptoms (PBC-40 cognitive domain score of ≥16), with 25 taking ursodeoxycholic acid (UDCA) (24 identified as biochemical responders according to Paris II criteria^46^), 5 intolerant to UDCA and 1 biochemical non-responder). All participants involved had fully compensated liver function throughout the intervention and stable baseline liver biochemistry (Table 2).

**Table 2:** Baseline characteristics.

Participant characteristics	Value
Female sex; n (%)	29 (97%)
Age at trial entry, years	52 (44-60)
Age at PBC diagnosis, years	46 (41-49)
White race, n (%)	28 (93%)
Weight, kg	73.1 (57.9-88.4)
BMI, kg/m^2^	27.6 (22.7-32.5)
MELD score	6 (6-8)
ALT, IU/L (ULN 55)	33 (24-67)
AST, IU/L (ULN 32)	41 (27-66)
Albumin, g/L (ULN 50)	42 (38-43)
ALP IU/L (ULN 130)	153 (125-218)
<1.9x ULN n (%)	24 (80%)
≥1.9x ULN n (%)	6 (20%)
Bilirubin, μmol/L (ULN 21)	12 (8-19)
GGT, IU/L (ULN 30)	105 (48-185)
Vitamin D, ng/ml (LLN 20)	57.2 (40.2-74.2)
TSH, mIU/ml (ULN 4.1)	1.34 (0.76-2.13)
Creatinine, μmol/L (ULN 104)	68 (58-75)
Haemoglobin, g/dl (ULN 16)	13.5 (12.4-14.2)
AMA positive, n (%)	22 (73%)
ANA positive, n (%)	4 (13%)
UDCA treated, n (%)	25 (81%)
UDCA intolerant, n (%)	5 (16%)
Second-line therapy∗	7 (23%)
Obeticholic acid	3
Fibric acid derivatives	3
Obeticholic acid/fibric acid therapy in combination	1
Biochemical response criteria met (Paris II), n (%)∗∗	24 (80%)
PBC-40 fatigue score	41 (37-43)
PBC Globe score	-0.73 (-1.9-0.77)
UK-PBC risk score 5 years (%)	1.39 (0.01-3.49)
UK-PBC risk score 10 years (%)	4.56 (0.04-11.22)
UK-PBC risk score 15 years (%)	8.32 (0.08-19.87)

ALT, alanine aminotransferase; AMA, anti-mitochondrial antibody; ANA, anti-nuclear antibody; AST, aspartate aminotransferase; GGT, gamma-glutamyltransferase; LLN, lower limit of normal; MELD, model for end-stage liver disease; PBC; primary biliary cholangitis; TSH, thyroid-stimulating hormone; UDCA, ursodeoxycholic acid; ULN, upper limit of normal.

∗ Of patients taking obeticholic acid as a second-line therapy at baseline, the dose was 5 mg daily in two patients, and 10 mg daily in two patients. Of patients taking fibric acid derivatives at baseline, all were taking bezafibrate at a dose of 400 mg daily.

∗∗ Biochemical response criteria were met in 17 patients treated with UDCA alone at baseline, in one participant treated with UDCA and obeticholic acid, one person taking UDCA and bezafibrate, two taking obeticholic acid alone, two taking bezafibrate alone, and in one individual taking obeticholic acid and bezafibrate without UDCA.

### Adverse events and safety

No serious adverse events were reported during the 12-week study period. One participant reported a transient increase in itch localised to the upper extremities (wrist) within 1 week of commencing the trial, which self-resolved without intervention. In addition, there were no significant changes in ALP (alkaline phosphatase), gamma-glutamyltransferase or other liver biochemical parameters within the study period.

### Adherence to HBEP

Overall, subjective adherence (*i.e*., 2-3 sessions completed per week) to the HBEP was 94% (29/31) in the first 6 weeks and 87% (27/31) following removal of the telehealth calls in the latter 6 weeks.

### Home-based exercise attenuates fatigue in PBC

**Fig. 2:**
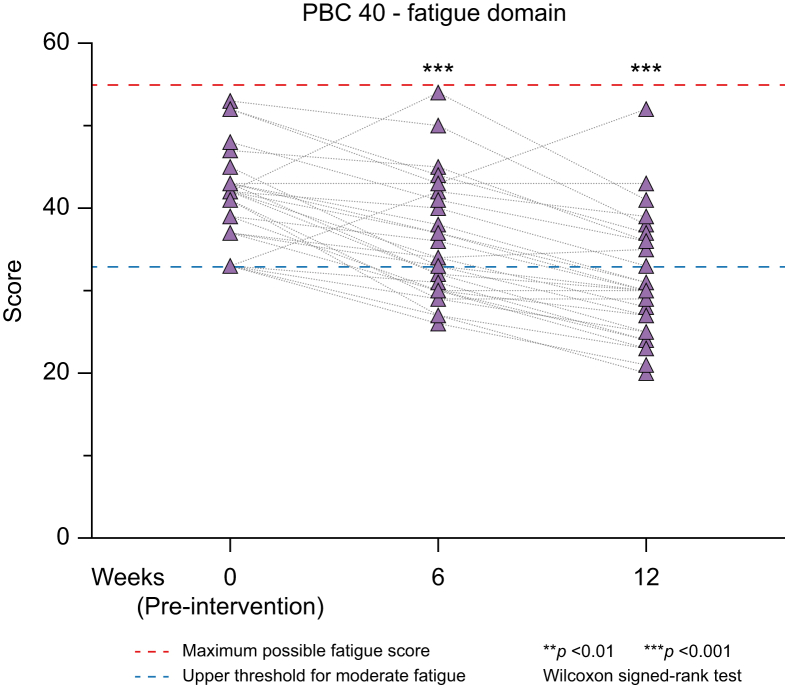
Effect of home-based exercise programme on PBC-associated fatigue. Changes in fatigue severity according to the PBC-40 score are shown from week 0 (baseline/pre-intervention) to week 6 and week 12 (end of study). Each individual point represents a patient at the given time, with broken lines showing their change in fatigue score. Wilcoxon signed ranked test was used to evaluate changes from baseline to week 6 and 12. ∗∗ denotes a *p* value less than 0.01, and ∗∗∗ a *p* value less than 0.001. PBC, primary biliary cholangitis.

The primary efficacy outcome measure was met in 26 participants with a median reduction of -10.5 (IQR -9 to -13; *p <*0.001) from baseline to week 12, with 22 individuals attaining PBC-40 fatigue domain scores below 33 (Fig. 2) (Table 3). On logistic regression analysis, there was no association between the odds of meeting the primary efficacy outcome measure and any of the baseline covariates (Table S2). No association was found between any of baseline UDCA treatment status, UDCA response, Global PBC score, UK-PBC score, or ALP values as a continuous or categorical measure, and the odds of meeting the primary outcome at 12 weeks.

**Table 3:** Effects of a home-based exercise program on PBC-40, health-related quality of life, sleep, and measures of physical function.

Outcomes	Baseline median scores	6-week median scores	6-week median changes∗	Z	*p* values	12-week median scores	12-week median changes∗	Z	*p* values
**PBC-40 domains (points)**									
**Total score**	131 (116.5-145.3)	113.5 (104-137.5)	-14 (-23 to -5.7)	-3.5	<0.001	102.5 (92.8-124)	-24 (-11.3 to -32.5)	-4.783	<0.001
Fatigue	41.5 (37-43)	34 (30-41)	-5 (-3 to -8)	-3.5	<0.001	30 (25-36)	-10.5 (-9 to -13)	-4.467	<0.001
Symptom	20 (16.8-22.3)	18 (15-20.3)	-2 (0.3 to -3)	-2.8	<0.004	16.5 (13-21)	-2 (-1 to -4)	-3.786	<0.01
Itch	8 (5-9.25)	6 (3-8)	-1 (0 to -2)	-3.9	0.001	5.5 (3-8)	-1 (0 to -2)	-3.209	0.001
Cognition	18.5 (14.5-22)	17.5 (13.5-20)	-2 (0 to -2.3)	-2.0	0.037	16.5 (11.5-18.3)	-3 (0 to -4)	-3.037	<0.01
Emotion	10 (8-13)	9 (6-11)	-1.5 (0 to -3)	-3.3	<0.001	8 (6-11)	-2 (-0.8 to -4)	-3.591	<0.01
Social	34.5 (27.8-37)	31 (27.75-35)	-1.5 (1 to - 4.2)	-2.2	0.027	30 (22-34)	-3.5 (-2 to -7.3)	3.424	<0.001
**DFIS (points)**	21 (17-24.3)	18.5 (12.75-21.5)	-4 (-1 to -5)	-2.4	<0.001	15.5 (10-18.5)	-6 (-4 to -8)	-3.534	<0.001

**HADS (points)**	17.5 (12.8-25.3)	16 (11-20.3)	-2 (-0.8 to -5)	-4.7	<0.001	15 (10-18.3)	-4 (-1 to -7.3)	-4.783	<0.001
Anxiety	9 (6-11.3)	8 (4.75-9)	-1 (-0.75 to -2.3)	-4.7	<0.001	6.5 (4-9)	-1 (-1 to -3)	-4.764	<0.001
Depression	9 (7-13)	8 (4.75-11)	-1 (0 to -3)	-0.4.4	<0.001	8 (4.8-10)	-2 (-0.3 to -4.3)	-4.508	<0.001
**ESS (points)**	14 (8.75-18)	12.5 (6.75-16)	-2 (0 to -3.3)	-2.6	<0.001	9 (5.8-12)	-4 (-3 to -6.3)	-4.641	<0.001
**Exercise/functional capacity**									

ISWT (m)	480 (357.5-622.5)	555 (407.5-723.5)	+50 (40-80)	4.3	<0.001	600 (427.5-755)	+90 (57.5-110)	4.765	<0.001
DASI (VO₂ peak ml/kg/min))	27.30 (20-29.7)	29.03 (21.9-34.6)	+1.64 (0-3.3)	3.7	<0.001	29.81 (24.1-34.6)	+2.41 (0.0-4.5)	4.088	<0.001
SPPB (points)	11 (10.8-12)	12 (12-12)	+1 (0-1)	3.8	<0.001	12 (12-12)	+1 (0-1.4)	3.743	<0.001
Chair stands	3 (3-4)	4 (4-4)	+0.5 (0-1)	3.6	<0.001	4 (4-4)	+1 (0-1.5)	3.666	<0.001
Balance	4 (4-4)	4 (4-4)	0 (0-0)	-1.0	0.317	4 (4-4)	0 (0-0)	0.000	1.000
Gait speed	4 (4-4)	4 (4-4)	0 (0-0)	0	1.000	4 (4-4)	0 (0-0)	1.890	0.059

DASI, Duke activity status index; DFIS, daily fatigue impact scale; ESS, Epworth sleepiness score; HADS, hospital anxiety and depression scale; ISWT, incremental shuttle walking test; SPPB, short physical performance battery.

∗ Median changes at 6 and 12 weeks calculated by averaging per-participant delta values taken between baseline and the indicated timepoint. Wilcoxon signed rank test was used to evaluate changes from baseline to week 6 and to week 12.

**Fig. 3:**
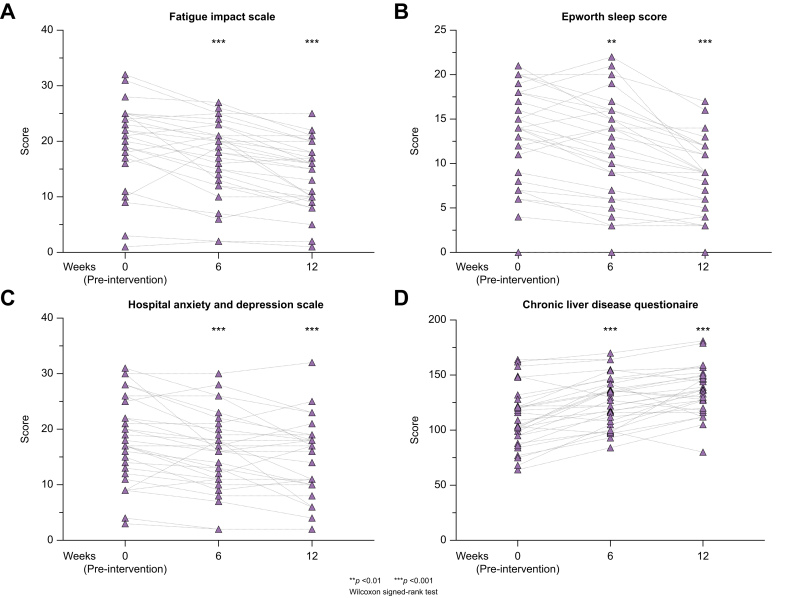
Effect of home-based exercise programme on broader domains of quality of life. Changes in specific domains of quality of life are shown according to (A) daily fatigue impact scale*,* (B) the Epworth sleepiness scale, (C) the hospital anxiety and depression scale*,* and (D) the chronic liver disease questionnaire. For each score, values are shown from week 0 (baseline/pre-intervention) to week 6 and week 12 (end of study). Each individual point represents a patient at the given time, with broken lines showing their change in score at subsequent time periods. Wilcoxon signed ranked test was used to evaluate changes from baseline to week 6 and 12. ∗∗ denotes a *p* value less than 0.01, and ∗∗∗ a *p* value less than 0.001.

Changes in fatigue severity were also observed according to the DFIS from baseline to week 6 (median reduction -4; IQR -1 to -5; *p <*0.001) and week 12 (median reduction -6; IQR, -4 to -8; *p <*0.001; Fig. 3), alongside the total PBC-40 score (median reduction -24; IQR -11.3 to -32.5; *p <*0.001) and cognitive, emotional, and overall symptom domains at week 12 (Table 3).

### Changes in daytime somnolence

Eleven participants reported excessive daytime somnolence (≥16 on ESS) at baseline, with a further 11 scoring 10-15. At 6 weeks, we observed a median change of -2 points on the ESS compared to baseline (IQR 0 to -3.3; *p <*0.001), and of -4 points at week 12 (IQR -3 to -6.3; *p <*0.001) (Fig. 3; Table 3). Among participants with a baseline ESS of ≥16 (n = 11), the median change in sleepiness score at week 12 was -4 points (IQR -3 to -6.5; *p <*0.001), with 9 of the 11 participants scoring ≤15.

### Associations between changes in fatigue severity, sleep and cognitive function

We observed a correlation between fatigue and daytime somnolence at baseline (r = 0.48, *p =* 0.007), and 6 weeks (r = 0.47, *p =* 0.009) and 12 weeks (r = 0.40, *p =* 0.03) post intervention, with daytime somnolence explaining 23%, 22% and 16% of the variation in fatigue score, respectively. Similarly, levels of anxiety and depression were lower from baseline to the aforementioned study time points, with a HADS score of 17.5 at baseline (IQR 12.8-25.3), 16 at 6 weeks (IQR 11-19.8; *p <*0.001), and 15 at 12 weeks (IQR 10-18.3; *p <*0.001; Fig. 3).

As fatigue scores lowered, self-reported cognitive symptoms also changed, with a median reduction of -2 points at week 6 (IQR 0 to -2.3; *p =* 0.038) and -3 at week 12 (IQR 0 to -4; *p <*0.020; Table 3). Additionally, there was a moderate correlation between fatigue and cognition at baseline, and at 6 and 12 weeks (r = 0.62 *p <*0.001; r = 0.52, *p =* 0.003; and r = 0.41, *p =* 0.023; respectively). We found that cognitive function explained 38%, 27% and 16% of the variation in fatigue scores, respectively.

In all, 16% of baseline, 34% of 6 week, and 52% of 12-week fatigue scores (PBC-40 fatigue domain) could not be explained by any of mood disorder, daytime somnolence or cognitive dysfunction.

### Aerobic exercise capacity and physical function

Changes in fatigue were seen in conjunction with increases (all *p <*0.001) in median ISWT (+90 m; IQR 57.5-110) and predicted VO₂ peak (+2.4 ml/kg/min; IQR 0.01-4.1) at 12 weeks. Additionally, 28/31 (90%) *vs*. 13/31 (43%) participants attained the maximum SPPB score of 12/12 (*p <*0.001) by 6 weeks, with chair stands contributing to the improvement seen at 6 and 12 weeks (Table 3). No correlation was found between fatigue severity scores and aerobic exercise capacity/physical function at baseline and 6 or 12 weeks post intervention.

## Discussion

Fatigue is an important and complex symptom, with significant impact on HRQoL, as reported in multiple PBC cohorts globally.^12^^,^^47^ Whilst therapeutic advances have led to improved life expectancy in PBC, symptom-directed treatments are limited with only modest reductions in fatigue severity.^7^^,^[48], [49], [50], [51], [52], [53] With up to 80% of the PBC population reporting fatigue, the emphasis to find a successful treatment is considered a priority amongst this cohort.^54^ Yet, little is offered to support individuals who suffer from fatigue, as rarely does this result in cost savings or reduced hospital admission rates. Moreover, data from the UK-PBC audit shows that more than one-third of patients are not even questioned about the presence of fatigue as part of routine clinical care.^55^ Herein, we show that a physiotherapy-led, individualised HBEP is safe and feasible. Whilst a single-centre, non-randomised trial, the 12-week programme was associated with a reduction in fatigue severity, increased physical function, and patient-reported improvements in symptom burden. Additionally, trial participants reported a reduction in daytime somnolence, including those scoring moderate-high on the ESS.

It is reported in healthy controls that repetitive exercise training can effect mechanisms that improve the muscular tolerance to prolonged exercise sessions and intensity.^56^^,^^57^ One such theory is that repetitive exercise influences both proton transporter function and blood flow, improving the kinetics of proton excretion from muscle.^57^^,^^58^ This, in turn, improves the muscles’ capacity to excrete acid and therefore its tolerance to longer exercise sessions. Findings within PBC suggest that repetitive exercise may mitigate pyruvate dehydrogenase complex dysfunction through activation of alternative pathways.^22^ Although our study offers no objective mechanistic data, this theory could offer one explanation for how participants were able to exercise for prolonged periods without negatively impacting on their levels of fatigue. Exercising at a moderate intensity has also been shown to impact on communication pathways between the central nervous system and peripheries regarding energy homeostasis. This has a potential positive impact on cognition and has also been linked to improved cellular energy metabolism with a reduction in oxidative stress and could offer a theory as to the observed changes in our study.^59^

The flexibility and supported approach to our exercise programme allowed patients to participate safely within their home environment, without the need for specialist equipment, allowing for greater autonomy. The use of remote monitoring and support in the early phase of the programme (weeks 0-6), provided through weekly telehealth calls, allowed for real time adaptation, problem solving, and the ability to monitor barriers to adherence. Despite withdrawal of telehealth calls at week 6, a reduction in fatigue severity continued to be observed until week 12. Thus, whilst specialist support is likely beneficial initially, participants may become confident in their own level of physical activity with less dependence on health-care professionals over time.

Changes in daytime somnolence and cognitive symptoms are also likely key to reducing fatigue in patients with PBC. One theory is that a combination of better sleep and improved mentation has the potential to improve central fatigue, by addressing abnormalities in cortical inhibitory/excitatory circuits.^33^ However, in the absence of objective testing this can only be speculated. Exercise interventions may also influence anxiety and depression, as reported in several chronic diseases.^60^^,^^61^ Across our cohort, the baseline prevalence of anxiety and depression was high according to the HADS.^62^ Whilst we observed an improvement in the HADS over time, scores remained within the ‘abnormal’ category at 12 weeks. This is perhaps unsurprising, given that targeted psychological support was not provided as part of our programme. Alongside studying exercise, greater understanding of the psychological needs of patients are required for a holistic ‘HRQoL-targeted’ intervention to be of value.^63^ Whilst it is not known by how much the ESS should fall to be considered clinically meaningful, a reduction of >25% and or to below a threshold of 10 have been proposed – both of which were observed following 12 weeks of HBEP.^64^^,^^65^

Our study is not without limitations, given the small sample size (hindered by emergence of the COVID-19 pandemic) and lack of a matched control group. By design, the HBEP was evaluated in a pilot feasibility study, and thus there is potential of selection bias. Secondly, individuals with hepatic decompensation and/or persistent jaundice were excluded. This is because as liver disease progresses, the pathogenesis of fatigue becomes more complex, with mechanisms that are not specific to disease aetiology, autoimmunity, or cholestasis. Additionally, even though adherence was subjectively maintained after withdrawal of weekly telehealth calls, the longevity of compliance needs to be explored beyond 12 weeks.

The PBC-40 HRQoL tool, whilst robustly validated in patients with PBC, is also not sensitive enough to discriminate between central and peripheral fatigue. Moreover, in the absence of muscle biopsies, calorimetry or magnetic resonance spectroscopy, the effects of exercise intervention on bioenergetics require further study in dedicated randomised trials. We must also acknowledge the intensity at which participants completed the HBEP, as this is open to subjective bias. Indeed, our programme relied on individuals self-reporting their perceived efforts, and despite education regarding work rate capacity, it is possible that participants exercised at lower intensities than reported. However, changes in aerobic and functional capacity suggest participants worked at a moderate intensity, but in the absence of close objective monitoring this can only be hypothesised.

It is also important to note that 19% of our study cohort were not being treated with UDCA due to reported intolerance. In part, this may be due to the principal trial centre being a tertiary liver unit, receiving referrals from many hospitals around the country, including of patients who are UDCA intolerant. Nevertheless, the rate of non-UDCA-treated patients is broadly reflective of the UK-PBC population as a whole,^3^ and similar to that captured in other countries.^66^ It is therefore important for future studies of exercise intervention to be conducted in larger group of patients, along with dedicated sub-group analysis of UDCA-treated *vs.* non-UDCA-treated individuals. Lastly, participants were not blinded to the primary aim of the intervention, which due to significant ethical concerns, and staunch objections from our patient and public involvement group, was not possible. This may have led to patients ‘expecting’ clinical benefit from study outset, leading to an overestimation of true treatment effect. Indeed, such phenomena have been observed in symptom studies of anti-itch therapy in PBC, wherein patients report subjective clinical benefit despite occupying the placebo arm of a randomised-controlled trial.^53^

Future randomised trials of exercise interventions need to be supported by efficacy and mechanistic evaluations, determining if (and how) HBEP benefits patients. Remote monitoring of physical activity through electronic applications or smart watch technology would also allow clinicians to monitor compliance, rather than relying on subjective patient diaries. As the impact of fatigue differs greatly between individuals, any intervention needs to offer a variety of exercises that could be progressed or regressed while still improving functional/aerobic capacity. Given the intervention phases, a broader choice of exercises may encourage longer term adherence in clinical practice and most importantly optimise patient autonomy.

In conclusion, our study highlights that a novel HBEP, incorporating straightforward low impact body-weighted exercise, is safe and feasible, and may have the potential to attenuate fatigue and daytime somnolence while improving functional capacity in PBC. These early findings may be applied to design future and larger controlled studies, to investigate the effects of HBEP on peripheral and central fatigue, alongside broader domains of HRQoL.

## Abbreviations

ESS, Epworth sleepiness score; HADS, hospital anxiety and depression scale; HBEP, home-based exercise programme; HRQoL, health-related quality of life; PBC, primary biliary cholangitis; RPE, rate of perceived exertion; SPPB, short physical performance battery; UDCA, ursodeoxycholic acid.

## Authors’ contributions

AF/FW: data extraction, study design, data and statistical analysis, writing of first draft of the manuscript including figures. AF/FW/SD: delivery of study intervention and reviewing/editing manuscript drafts through to submission. JH/PJT/AF: participant identification and reviewing/editing manuscript drafts through to submission. MJA/PJT: study concept and design, data and statistical analysis, revision of manuscript drafts through to submission (and resubmission). PJT: Study conception and guarantor of the article.

## Data availability statement

Data is available upon request from the corresponding author.

## Financial support

Palak J. Trivedi receives institutional salary support from the National Institute for Health Research (NIHR) Birmingham Biomedical Research Centre (BRC). This paper presents independent research supported by the Birmingham NIHR BRC based at the University Hospitals Birmingham National Health Service (NHS) Foundation Trust and the University of Birmingham. The views expressed are those of the author(s) and not necessarily those of the NHS, the NIHR, or the Department of Health. This paper presents independent research supported by the BRC based at the University Hospitals Birmingham NHS Foundation Trust and the University of Birmingham. This study was supported by an unrestricted grant provided by Intercept Pharmaceuticals.

## Conflicts of interest

The authors of this study declare that they do not have any conflict of interest.

Please refer to the accompanying ICMJE disclosure forms for further details.
