# Supplementary material for: A home-based exercise programme attenuates fatigue in primary biliary cholangitis: Results from the EXCITED clinical trial
Source: JHEP Rep. 2024 Sep 6;6(12):101210. doi: 10.1016/j.jhepr.2024.101210 (PMC11617285; doi:10.1016/j.jhepr.2024.101210)
Supplement: Multimedia component 4 [file mmc4.pdf]

A home-based exercise programme attenuates fatigue in primary biliary cholangitis: Results from the EXCITED clinical trial

Alice Freer[1,2,*,†], **Felicity R. Williams**[1,2,3,†], Simon Durman[2], Jennifer Hayden[2], Matthew J. Armstrong[1,2,*], Palak J. Trivedi[1,2,4,5,*]

JHEP Reports **2024**. vol. 6 | 1–10

Check for updates

Background & Aims: Fatigue is a commonly reported symptom of primary biliary cholangitis (PBC). We conducted a single-arm, open-label clinical trial to assess the efficacy of a physiotherapist-led home-based exercise programme (HBEP) in patients with PBC and moderate-to-severe fatigue (NCT04265235).

Methods: A 12-week individualised HBEP (aerobic + resistance based) was delivered to patients with a PBC-40 fatigue domain score ≥33. The primary efficacy outcome measure was a reduction in fatigue severity by ≥5 points. Secondary outcome measures included other domains of PBC-40, the FIS (fatigue impact scale), ESS (Epworth sleepiness score), HADS (hospital anxiety and depression scale), aerobic capacity (ISWT [incremental shuttle walk test], Duke activity status index (predicted VO_2 peak) and physical function (short physical performance battery [SPPB]).

Results: A total of 31 patients were recruited, of whom 30 completed the 12-week HBEP (29 women; median age 53 years, median alkaline phosphatase value: 1.5x the upper limit of normal, median bilirubin: 12 µmol/L, and median baseline PBC-40 fatigue score 42). The primary outcome was met by 26 patients, with a median reduction in PBC-40 fatigue score of -10.5 points (IQR -9 to -13; p <0.001). Reductions were also observed in the symptom, cognition, and emotion domains of PBC-40, and in the FIS, ESS and HADS (p <0.01 for all measures). This was alongside increases in the median ISWT (+90 m; IQR 57.5-110), predicted VO_2 peak (+2.41 ml/kg/min; IQR 0.01-4.05), and SPPB (+1 point; IQR 0-1.4) (all p <0.001). 28 participants achieved the maximum SPPB score of 12/12 (*vs.* 13 patients at baseline; p <0.001). No significant adverse events were reported.

Conclusion: This proof-of-concept study shows that a HBEP is safe, feasible, and has the potential to attenuate fatigue. Controlled trials are needed to validate the efficacy of exercise interventions in PBC.

© 2024 The Authors. Published by Elsevier B.V. on behalf of European Association for the Study of the Liver (EASL). This is an open access article under the CC BY license (http://creativecommons.org/licenses/by/4.0/).

Introduction

Primary biliary cholangitis (PBC) is a rare chronic cholestatic disease, in which clinical outcomes are dictated by progression to cirrhosis and need for transplantation.[1,2] Effective bile acid therapy is associated with improved transplant-free survival and lower rates of liver cancer, but with limited impact on symptom burden.[3–8] Whilst considered a rare disease, the impact on patient quality of life is substantial, with symptoms dominated by pruritus, cognitive impairment, and fatigue.[9–12] The latter can be severe and debilitating, and is reported by 40-80% of patients across the UK-PBC population.[3] Whilst fatigue is not an indicator of liver disease severity, it is associated with heightened mortality risk.[13,14] Moreover, there are no effective pharmacological interventions for fatigue in PBC, and >40% of patients have persistent and significant symptoms following liver transplantation.[15–17]

Fatigue in PBC is not thought to be the result of a single activity or challenging functional task, but rather an inability to perform sustained activities or movements over time. The cause of fatigue in cholestasis is likely to be multifactorial, with contributions from both central (*i.e.* decrease in voluntary activation of muscle relating to a decline in motor cortex drive) and peripheral factors (*i.e.* decrease in contractile strength and endurance relating to altered muscle action potential mechanisms).[18–25] Peripherally, muscle bioenergetic abnormalities, alongside suppressed anaerobic threshold during physical exertion, have been proposed.[22,23] For example, when compared to healthy controls, patients with PBC-associated fatigue manifest delayed peripheral muscle pH recovery and excessive acidosis following ≤35% maximal voluntary contraction of lower leg muscles.[22] Despite this, mechanistic studies show that repeated exercise can

* Corresponding authors. Address: NIHR Birmingham BRC, Institute of Immunology and Immunotherapy, University of Birmingham, B15 2TT; UK; (P.J. Trivedi), or (A. Freer).
E-mail addresses: p.j.trivedi@bham.ac.uk (P.J. Trivedi), alice.freer@uhb.nhs.uk (A. Freer).
† Denotes joint first authorship
* Denotes joint senior authorship
https://doi.org/10.1016/j.jhepr.2024.101210

attenuate peripheral discrepancies, which may improve exercise tolerance.[22]

Exercise intervention has been shown to attenuate central fatigue in healthy individuals and those with long-term neurological, respiratory, and oncological conditions.[23,26–32] This may also be relevant to individuals with PBC, who concomitantly report memory and cognitive decline, increased daytime somnolence and low mood, attributed to abnormalities in central activation of cortical inhibitory and excitatory circuits, resulting in central nervous system dysfunction.[33] The use of repetitive/progressive exercise programmes at a moderate intensity (safe zone training) allows for steady adaptation at a muscular level, and improvement in exercise tolerance through physiological acclimation.[34] This, combined with the hypothesis that repeated exercise may attenuate altered muscle bioenergetics, or improve central processes, offers an opportunity to explore the impact of a structured, moderately intense exercise programme on fatigue in cholestasis.

The overarching goal of the study was to conduct an open-label clinical trial to evaluate the impact of a physiotherapy-led exercise initiative, specifically to attenuate fatigue associated with PBC. Importantly, this was a home-based exercise programme (HBEP), designed specifically to overcome logistical, travel and cost challenges associated with a hospital-based setting.

Patients and methods

A single-arm (open-label) trial was designed to investigate the impact and safety of a 12-week HBEP; specifically on fatigue, health-related quality of life (HRQoL), sleep quality, aerobic exercise capacity and physical function, in patients with PBC. Ethical approval was obtained from the National Research Ethics Service Committee in London and the Health Research Authority (Research Ethics Service reference number 18/LO/2109) and was approved by the Queen Elizabeth University Hospital Birmingham Research and Development Department. The study was registered with clinicaltrials.gov (NC04265235).

Participant identification and eligibility

Detailed descriptors of the full study protocol and trial schema, including specific inclusion/exclusion criteria, the natur.e of patient and public involvement and the specific analysis plan are presented elsewhere (Supplementary File 1 [35] and Fig. S1). Briefly, as part of the Birmingham symptom-focussed specialist nurse programme, all individuals who attend PBC outpatient clinics complete a PBC-40 quality-of-life questionnaire as part of routine standard of care during outpatient clinic visits. This allowed timely identification and pre-screening of eligible participants with moderate-to-severe fatigue, as evidenced by a score of ≥33 in the fatigue domain of PBC-40 (a validated health-related quality-of-life tool for patients with PBC).[15,35,36]

Prior to starting any intervention, patients attended a dedicated screening visit (2 weeks after pre-screening indicated trial eligibility). This was to ensure stability in symptom severity, as indicated by persistently elevated PBC-40 fatigue scores ≥33 between visits. Individuals with additional contributors to fatigue were excluded; encompassing (but not limited to) those with anaemia (any cause), renal dysfunction, concomitant malignancy, untreated or poorly treated thyroid disease, other untreated or poorly treated extrahepatic autoimmune diseases, vitamin B or D deficiency, gluten-sensitive enteropathy, and untreated or poorly treated diabetes mellitus. Other key exclusion criteria were decompensated cirrhosis +/- portal hypertension, serum bilirubin values >50 µmol/L (in the absence of Gilberts syndrome), prior solid organ transplantation, a diagnosis of autonomic dysfunction, and/or cardiovascular instability.

Intervention

Informed written consent was obtained from all participants prior to study entry. All participants underwent a baseline (visit 1) evaluation of aerobic exercise capacity/functional capacity (incremental shuttle walking test [ISWT], Duke activity status index [DASI]) and physical function (short physical performance battery [SPPB]) by a specialist liver physiotherapist.[37–39] These outcomes, alongside the participant's confidence to complete the exercise guided the design of their individualised resistance programme; specifically, their entry level and number of sets (Table 1). To ensure a moderate intensity work rate was achieved, participants were educated on the Borg rate of perceived exertion (RPE) and provided a hand out with clear colour-coded training zones (white a prompt to increase intensity, green being optimal (RPE 12-14), amber a prompt to reduce intensity, and red to highlight their work effort was too high) (Fig. S2).[40]

Prior to leaving the baseline visit, each participant completed their exercise programme under the supervision of the specialist, hospital-based liver physiotherapist. The latter is defined as an experienced clinician with a Bachelor of Science degree in Physiotherapy, with the ability to assess, treat, manage and prescribe advice on exercise prescription, fatigue management and lifestyle advice for patients with complex liver disease, ensuring the desired exercise intensity is possible to achieve. Importantly, it was essential that the physiotherapist had at least 12 months' experience of working in a tertiary liver unit. Participants were then provided with a diary to record adherence/compliance and asked to complete their HBEP 2-3 times per week for 12 weeks. Importantly, the HBEPs were designed to be low impact and in a circuit format, without need for specialist equipment.

Between weeks 1-5 (inclusive) of intervention, the specialist liver physiotherapist provided weekly structured 'virtual' telehealth consultations to each participant. These consultations evaluated participant engagement in the HBEP, highlighted limiting factors (such as mood) which may be influencing adherence, and whether any new symptoms of concern such as syncope, muscular pain or chest pain occurred. Following this discussion, the HBEP was reviewed with the participant. Dependent on responses to (a) confidence completing the programme, (b) ability to elicit a work rate of 12-14 using the RPE, (c) frequency of self-reported days with fatigue, the programme was progressed following each telehealth call (*i.e.*, reducing rest time between work phases/sets) or regressed (*i.e.*, increasing rest time between work phases/sets) between levels (Fig. 1). This was guided by patient fatigue to ensure that individuals were not overwhelmed by the HBEP.

Participants next attended the trial site at week 6 (visit 2) for clinical review and physical examination, followed by a further 6 weeks of HBEP (weeks 7 to 11) wherein the routine weekly telehealth consultations were withdrawn. A final study visit with clinical review was conducted at week 12 (visit 3). Participants

Table 1. Levels of exercise with suggested work rate, rest phases and number of sets.

Level	Exercises	Work phase	Rest phase	Number of sets per exercise
1	Sit to stand<br>Bench press<br>Frog squat<br>Bear crawl<br>Step-up	20 s	40 s	3-5
2	Sit to stand<br>Bench press<br>Frog squat<br>Bear crawl<br>Step-up	30 s	30 s	3-5
3	Sit to stand<br>Frog squat<br>Bear crawl<br>Kick sit<br>Body drop	30 s	30 s	4-5
4	Frog squat<br>Bear crawl<br>Kick sit<br>Body drop<br>Cobra<br>Polar press	30 s	30 s	4-5
5	Frog squat<br>Bear crawl<br>Kick sit<br>Body drops<br>Cobra<br>Polar press	40 s	20 s	5

were not limited from making other changes to their lifestyle (including dietary requirements) and were given standard healthy dietary advice throughout. Patients were advised to cease the programme if any adverse effects developed, such as chest discomfort, refractory itch, or syncope, and contact the trial team immediately.

Outcome measures

The primary outcome measure was the proportion of patients attaining a reduction in PBC-40 fatigue domain score of ≥5 points at 12 weeks.[15,35,41] The PBC-40 fatigue domain is widely accepted as an endpoint in HRQoL clinical trials, although there is lack of consensus as to what constitutes a minimum important change. Thus, our primary outcome measure was largely informed by prior interventional studies seeking to improve fatigue in PBC.[15,41] Primarily, ours was an open-label pilot study, with the overarching goal of seeing if a HBEP was feasible, deliverable, and associated with a reduction in fatigue severity over a 12-week period. As such, the sample size was chosen pragmatically, based on published UK-PBC data, and powered to detect a mean change in the PBC-40 fatigue domain score of 5 units at 12 weeks (a difference associated with significantly greater levels of social function).[11] Previous studies have shown a standard deviation of 8 units and a correlation of 0.6 between baseline and follow-up. Thus, using a power of 90% and significance level of 5% would require outcome data from 35 participants for an interventional arm in a 1:1 randomised-controlled trial, including a 10% attrition rate (if power was lowered to 80% because PBC is a rare disease, this would be 29 patients per arm). Thus, our intended sample size was a total of 40 patients for a single-arm study.

Key secondary outcome measures included the median change from baseline in the DFIS (daily fatigue impact scale),[42] other domains of PBC-40 (emotional, social, and cognitive function, general symptoms and itch),[36] the Epworth sleepiness score (ESS),[43] the hospital anxiety and depression scale (HADS),[44] impact fatigue had on cognitive function, score changes in the chronic liver disease questionnaire,[45] and measures of aerobic exercise/functional capacity (incremental shuttle walking test [ISWT], Duke activity status index [DASI]) and physical function (SPPB [short physical performance battery]). All outcome measures were recorded at baseline, week 6 and week 12.

Data analysis

Baseline characteristics were analysed using descriptive statistics and expressed as median (IQR), and n (%) for categorical data. Fisher's exact test was used to analyse differences between categorical variables, and the Mann-Whitney *U* test for analysing differences between two groups of unpaired continuous variables. Wilcoxon signed ranked test was used to evaluate changes in continuous variables from baseline to week 6 and 12. Bonferroni-Dunn *post hoc* correction analysis was performed when performing multiple test evaluations on a single set of data. Logistic regression analysis was performed to identify factors associated with a ≥5 point reduction in the PBC-40 fatigue domain. Following completion of a Shapiro-Wilk's test ($p > 0.05$), a Pearson's product-moment correlation was also used to assess the relationship between baseline, and post-intervention measures at weeks 6 and 12.

Results

Pre-screening and characteristics of the studied cohort

The study was conducted between April 2019 to March 2020. Of 261 pre-identified patients, 148 recorded a PBC-40 fatigue score ≥33, of whom 78 met study eligibility criteria. Thirty-four patients passed screening and agreed to take part, but due to emergence of the first wave of the COVID-19 pandemic, trial entry ceased after the 31st consecutive participant was recruited. One participant withdrew from the study prior to week 6 due to unforeseen personal circumstances (Fig. S3). No significant differences were observed between trial eligible participants and those who were deemed eligible on pre-screening but who declined to take part in the study (Table S1).

Of the 30/31 participants who completed the 12-week HBEP, 97% were women, with a median age of 52 years (IQR 44-60 years) and PBC-40 fatigue domain score of 41.5 (IQR 37-43) (Table 2). Twenty-two participants were classified as having at least moderate cognitive symptoms (PBC-40 cognitive domain score of ≥16), with 25 taking ursodeoxycholic acid (UDCA) (24 identified as biochemical responders according to Paris II criteria[46]), 5 intolerant to UDCA and 1 biochemical non-responder). All participants involved had fully compensated liver function throughout the intervention and stable baseline liver biochemistry (Table 2).

Adverse events and safety

No serious adverse events were reported during the 12-week study period. One participant reported a transient increase in itch localised to the upper extremities (wrist) within 1 week of

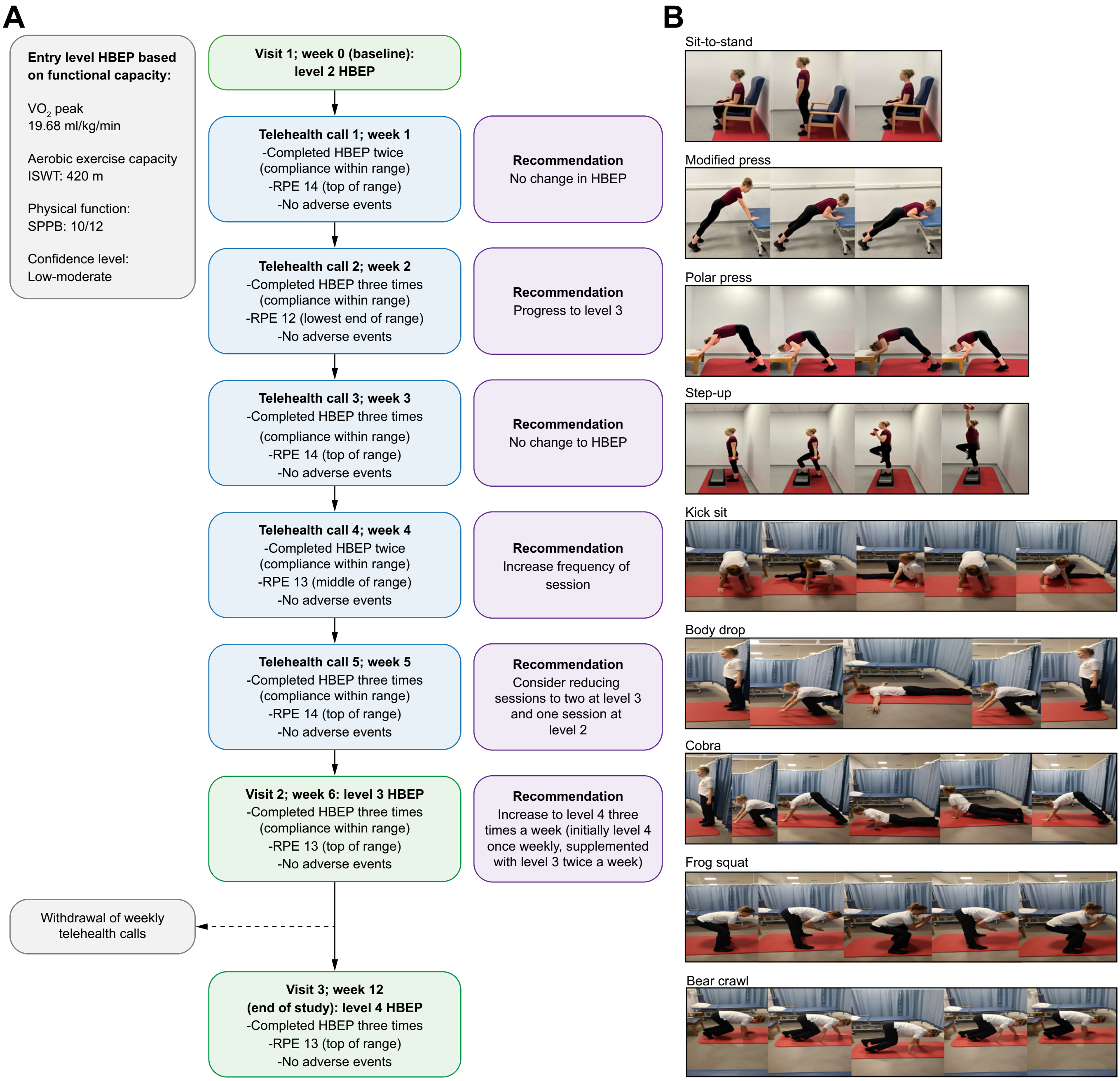

Fig. 1. Representative exercise prescription. (A) An example of the initial exercise prescription based on functional capacity, aerobic exercise capacity, physical function and participant confidence level, progression/regression of programme based on compliance and work rate, following weekly telehealth calls between visits 1 and 2, and alterations of programme at visit 2 prior to routine scheduled telehealth calls being removed between visits 2 and 3. (B) Is indicative of resistance exercises that comprise the exercise prescription. HBEP, home-based exercise programme; ISWT, incremental shuttle walk test; RPE, rate of perceived exertion; SPPB, short performance physical battery.

commencing the trial, which self-resolved without intervention. In addition, there were no significant changes in ALP (alkaline phosphatase), gamma-glutamyltransferase or other liver biochemical parameters within the study period.

Adherence to HBEP

Overall, subjective adherence (*i.e.*, 2-3 sessions completed per week) to the HBEP was 94% (29/31) in the first 6 weeks and 87% (27/31) following removal of the telehealth calls in the latter 6 weeks.

Home-based exercise attenuates fatigue in PBC

The primary efficacy outcome measure was met in 26 participants with a median reduction of -10.5 (IQR -9 to -13; p <0.001) from baseline to week 12, with 22 individuals attaining PBC-40 fatigue domain scores below 33 (Fig. 2) (Table 3). On logistic regression analysis, there was no association between the odds of meeting the primary efficacy outcome measure and any of the baseline covariates (Table S2). No association was found between any of baseline UDCA treatment status, UDCA response, Global PBC score, UK-PBC score, or ALP values as

a continuous or categorical measure, and the odds of meeting the primary outcome at 12 weeks.

Changes in fatigue severity were also observed according to the DFIS from baseline to week 6 (median reduction -4; IQR -1 to -5; *p* <0.001) and week 12 (median reduction -6; IQR, -4 to -8; *p* <0.001; Fig. 3), alongside the total PBC-40 score (median reduction -24; IQR -11.3 to -32.5; *p* <0.001) and cognitive, emotional, and overall symptom domains at week 12 (Table 3).

Changes in daytime somnolence

Eleven participants reported excessive daytime somnolence (≥16 on ESS) at baseline, with a further 11 scoring 10-15. At 6 weeks, we observed a median change of -2 points on the ESS compared to baseline (IQR 0 to -3.3; *p* <0.001), and of -4 points at week 12 (IQR -3 to -6.3; *p* <0.001) (Fig. 3; Table 3). Among participants with a baseline ESS of ≥16 (n = 11), the median change in sleepiness score at week 12 was -4 points (IQR -3 to -6.5; *p* <0.001), with 9 of the 11 participants scoring ≤15.

Table 2. Baseline characteristics.

Participant characteristics	Value
Female sex; n (%)	29 (97%)
Age at trial entry, years	52 (44-60)
Age at PBC diagnosis, years	46 (41-49)
White race, n (%)	28 (93%)
Weight, kg	73.1 (57.9-88.4)
BMI, kg/m^2	27.6 (22.7-32.5)
MELD score	6 (6-8)
ALT, IU/L (ULN 55)	33 (24-67)
AST, IU/L (ULN 32)	41 (27-66)
Albumin, g/L (ULN 50)	42 (38-43)
ALP IU/L (ULN 130)	153 (125-218)
<1.9x ULN n (%)	24 (80%)
≥1.9x ULN n (%)	6 (20%)
Bilirubin, µmol/L (ULN 21)	12 (8-19)
GGT, IU/L (ULN 30)	105 (48-185)
Vitamin D, ng/ml (LLN 20)	57.2 (40.2-74.2)
TSH, mIU/ml (ULN 4.1)	1.34 (0.76-2.13)
Creatinine, µmol/L (ULN 104)	68 (58-75)
Haemoglobin, g/dl (ULN 16)	13.5 (12.4-14.2)
AMA positive, n (%)	22 (73%)
ANA positive, n (%)	4 (13%)
UDCA treated, n (%)	25 (81%)
UDCA intolerant, n (%)	5 (16%)
Second-line therapy*	7 (23%)
Obeticholic acid	3
Fibric acid derivatives	3
Obeticholic acid/fibric acid therapy in combination	1
Biochemical response criteria met (Paris II), n (%)**	24 (80%)
PBC-40 fatigue score	41 (37-43)
PBC Globe score	-0.73 (-1.9-0.77)
UK-PBC risk score 5 years (%)	1.39 (0.01-3.49)
UK-PBC risk score 10 years (%)	4.56 (0.04-11.22)
UK-PBC risk score 15 years (%)	8.32 (0.08-19.87)

ALT, alanine aminotransferase; AMA, anti-mitochondrial antibody; ANA, anti-nuclear antibody; AST, aspartate aminotransferase; GGT, gamma-glutamyltransferase; LLN, lower limit of normal; MELD, model for end-stage liver disease; PBC; primary biliary cholangitis; TSH, thyroid-stimulating hormone; UDCA, ursodeoxycholic acid; ULN, upper limit of normal.

*Of patients taking obeticholic acid as a second-line therapy at baseline, the dose was 5 mg daily in two patients, and 10 mg daily in two patients. Of patients taking fibric acid derivatives at baseline, all were taking bezafibrate at a dose of 400 mg daily.

**Biochemical response criteria were met in 17 patients treated with UDCA alone at baseline, in one participant treated with UDCA and obeticholic acid, one person taking UDCA and bezafibrate, two taking obeticholic acid alone, two taking bezafibrate alone, and in one individual taking obeticholic acid and bezafibrate without UDCA.

Associations between changes in fatigue severity, sleep and cognitive function

We observed a correlation between fatigue and daytime somnolence at baseline (r = 0.48, *p* = 0.007), and 6 weeks (r = 0.47, *p* = 0.009) and 12 weeks (r = 0.40, *p* = 0.03) post intervention, with daytime somnolence explaining 23%, 22% and 16% of the variation in fatigue score, respectively. Similarly, levels of anxiety and depression were lower from baseline to the aforementioned study time points, with a HADS score of 17.5 at baseline (IQR 12.8-25.3), 16 at 6 weeks (IQR 11-19.8; *p* <0.001), and 15 at 12 weeks (IQR 10-18.3; *p* <0.001; Fig. 3).

As fatigue scores lowered, self-reported cognitive symptoms also changed, with a median reduction of -2 points at week 6 (IQR 0 to -2.3; *p* = 0.038) and -3 at week 12 (IQR 0 to -4; *p* <0.020; Table 3). Additionally, there was a moderate correlation between fatigue and cognition at baseline, and at 6 and 12 weeks (r = 0.62 *p* <0.001; r = 0.52, *p* = 0.003; and r = 0.41, *p* = 0.023; respectively). We found that cognitive function explained 38%, 27% and 16% of the variation in fatigue scores, respectively.

In all, 16% of baseline, 34% of 6 week, and 52% of 12-week fatigue scores (PBC-40 fatigue domain) could not be explained by any of mood disorder, daytime somnolence or cognitive dysfunction.

Aerobic exercise capacity and physical function

Changes in fatigue were seen in conjunction with increases (all *p* <0.001) in median ISWT (+90 m; IQR 57.5-110) and predicted VO_2 peak (+2.4 ml/kg/min; IQR 0.01-4.1) at 12 weeks. Additionally, 28/31 (90%) *vs.* 13/31 (43%) participants attained the maximum SPPB score of 12/12 (*p* <0.001) by 6 weeks, with chair stands contributing to the improvement seen at 6 and 12 weeks (Table 3). No correlation was found between fatigue

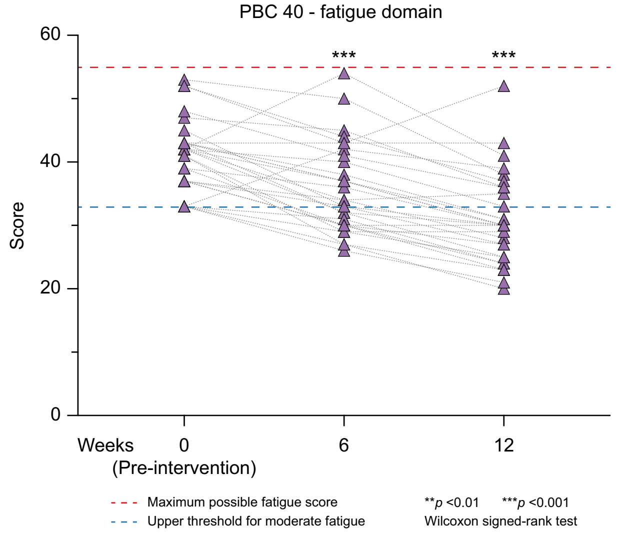

Fig. 2. Effect of home-based exercise programme on PBC-associated fatigue. Changes in fatigue severity according to the PBC-40 score are shown from week 0 (baseline/pre-intervention) to week 6 and week 12 (end of study). Each individual point represents a patient at the given time, with broken lines showing their change in fatigue score. Wilcoxon signed ranked test was used to evaluate changes from baseline to week 6 and 12. ** denotes a *p* value less than 0.01, and *** a *p* value less than 0.001. PBC, primary biliary cholangitis.

severity scores and aerobic exercise capacity/physical function at baseline and 6 or 12 weeks post intervention.

Discussion

Fatigue is an important and complex symptom, with significant impact on HRQoL, as reported in multiple PBC cohorts globally.[12,47] Whilst therapeutic advances have led to improved life expectancy in PBC, symptom-directed treatments are limited with only modest reductions in fatigue severity.[7,48–53] With up to 80% of the PBC population reporting fatigue, the emphasis to find a successful treatment is considered a priority amongst this cohort.[54] Yet, little is offered to support individuals who suffer from fatigue, as rarely does this result in cost savings or reduced hospital admission rates. Moreover, data from the UK-PBC audit shows that more than one-third of patients are not even questioned about the presence of fatigue as part of routine clinical care.[55] Herein, we show that a physiotherapy-led, individualised HBEP is safe and feasible. Whilst a single-centre, non-randomised trial, the 12-week programme was associated with a reduction in fatigue severity, increased physical function, and patient-reported improvements in symptom burden. Additionally, trial participants reported a reduction in daytime somnolence, including those scoring moderate-high on the ESS.

It is reported in healthy controls that repetitive exercise training can effect mechanisms that improve the muscular tolerance to prolonged exercise sessions and intensity.[56,57] One such theory is that repetitive exercise influences both proton transporter function and blood flow, improving the kinetics of proton excretion from muscle.[57,58] This, in turn, improves the muscles' capacity to excrete acid and therefore its tolerance to longer exercise sessions. Findings within PBC suggest that repetitive exercise may mitigate pyruvate dehydrogenase complex dysfunction through activation of alternative pathways.[22] Although our study offers no objective mechanistic data, this theory could offer one explanation for how participants were able to exercise for prolonged periods

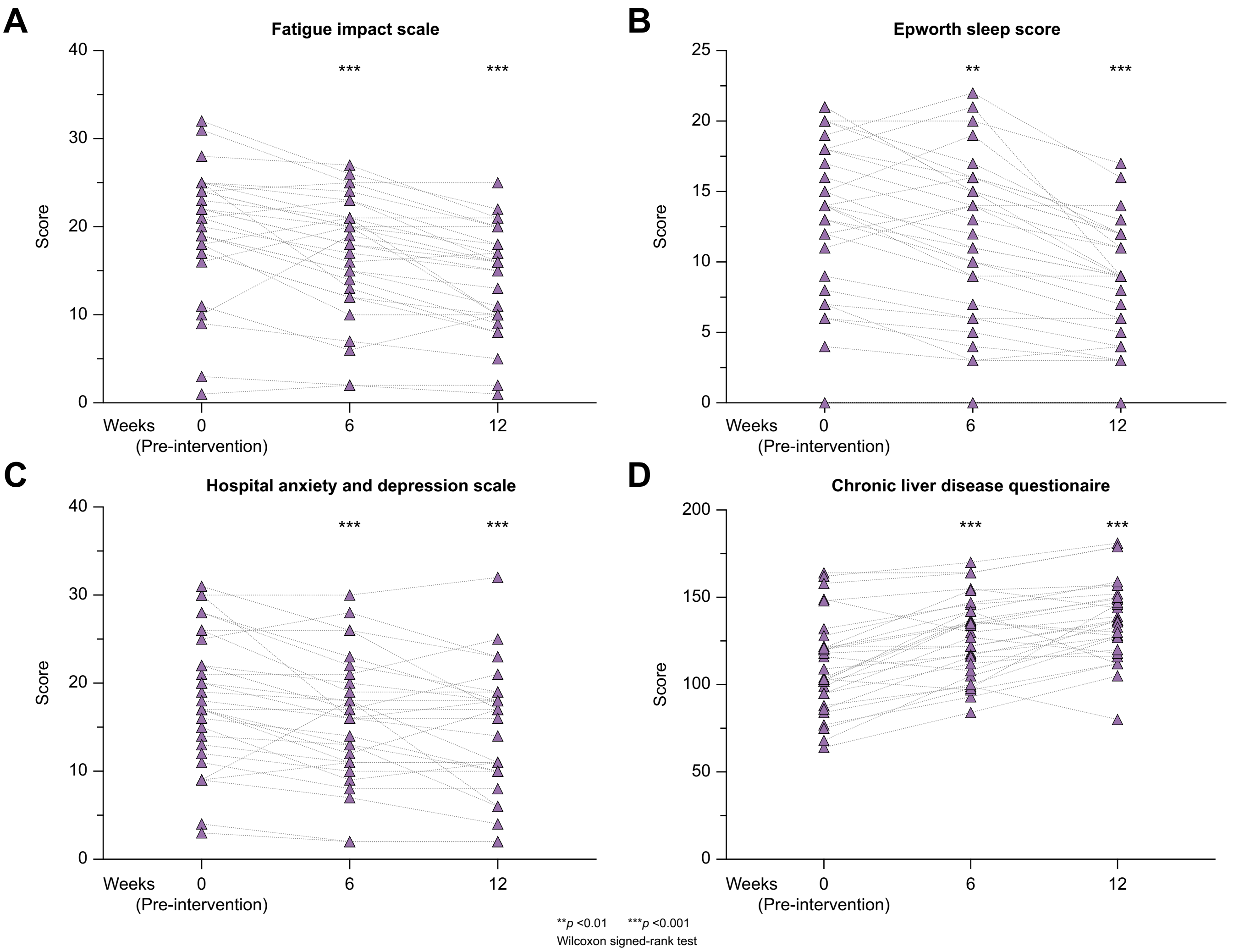

Fig. 3. Effect of home-based exercise programme on broader domains of quality of life. Changes in specific domains of quality of life are shown according to (A) daily fatigue impact scale, (B) the Epworth sleepiness scale, (C) the hospital anxiety and depression scale, and (D) the chronic liver disease questionnaire. For each score, values are shown from week 0 (baseline/pre-intervention) to week 6 and week 12 (end of study). Each individual point represents a patient at the given time, with broken lines showing their change in score at subsequent time periods. Wilcoxon signed ranked test was used to evaluate changes from baseline to week 6 and 12. ** denotes a p value less than 0.01, and *** a p value less than 0.001.

Table 3. Effects of a home-based exercise program on PBC-40, health-related quality of life, sleep, and measures of physical function.

Outcomes	Baseline median scores	6-week median scores	6-week median changes*	Z	*p* values	12-week median scores	12-week median changes*	Z	*p* values
**PBC-40 domains (points)**									
**Total score**	131 (116.5-145.3)	113.5 (104-137.5)	-14 (-23 to -5.7)	-3.5	<0.001	102.5 (92.8-124)	-24 (-11.3 to -32.5)	-4.783	<0.001
Fatigue	41.5 (37-43)	34 (30-41)	-5 (-3 to -8)	-3.5	<0.001	30 (25-36)	-10.5 (-9 to -13)	-4.467	<0.001
Symptom	20 (16.8-22.3)	18 (15-20.3)	-2 (0.3 to -3)	-2.8	<0.004	16.5 (13-21)	-2 (-1 to -4)	-3.786	<0.01
Itch	8 (5-9.25)	6 (3-8)	-1 (0 to -2)	-3.9	0.001	5.5 (3-8)	-1 (0 to -2)	-3.209	0.001
Cognition	18.5 (14.5-22)	17.5 (13.5-20)	-2 (0 to -2.3)	-2.0	0.037	16.5 (11.5-18.3)	-3 (0 to -4)	-3.037	<0.01
Emotion	10 (8-13)	9 (6-11)	-1.5 (0 to -3)	-3.3	<0.001	8 (6-11)	-2 (-0.8 to -4)	-3.591	<0.01
Social	34.5 (27.8-37)	31 (27.75-35)	-1.5 (1 to - 4.2)	-2.2	0.027	30 (22-34)	-3.5 (-2 to -7.3)	3.424	<0.001
**DFIS (points)**	21 (17-24.3)	18.5 (12.75-21.5)	-4 (-1 to -5)	-2.4	<0.001	15.5 (10-18.5)	-6 (-4 to -8)	-3.534	<0.001
**HADS (points)**	17.5 (12.8-25.3)	16 (11-20.3)	-2 (-0.8 to -5)	-4.7	<0.001	15 (10-18.3)	-4 (-1 to -7.3)	-4.783	<0.001
Anxiety	9 (6-11.3)	8 (4.75-9)	-1 (-0.75 to -2.3)	-4.7	<0.001	6.5 (4-9)	-1 (-1 to -3)	-4.764	<0.001
Depression	9 (7-13)	8 (4.75-11)	-1 (0 to -3)	-0.4.4	<0.001	8 (4.8-10)	-2 (-0.3 to -4.3)	-4.508	<0.001
**ESS (points)**	14 (8.75-18)	12.5 (6.75-16)	-2 (0 to -3.3)	-2.6	<0.001	9 (5.8-12)	-4 (-3 to -6.3)	-4.641	<0.001
**Exercise/functional capacity**									
ISWT (m)	480 (357.5-622.5)	555 (407.5-723.5)	+50 (40-80)	4.3	<0.001	600 (427.5-755)	+90 (57.5-110)	4.765	<0.001
DASI (VO_2 peak ml/kg/min))	27.30 (20-29.7)	29.03 (21.9-34.6)	+1.64 (0-3.3)	3.7	<0.001	29.81 (24.1-34.6)	+2.41 (0.0-4.5)	4.088	<0.001
SPPB (points)	11 (10.8-12)	12 (12-12)	+1 (0-1)	3.8	<0.001	12 (12-12)	+1 (0-1.4)	3.743	<0.001
Chair stands	3 (3-4)	4 (4-4)	+0.5 (0-1)	3.6	<0.001	4 (4-4)	+1 (0-1.5)	3.666	<0.001
Balance	4 (4-4)	4 (4-4)	0 (0-0)	-1.0	0.317	4 (4-4)	0 (0-0)	0.000	1.000
Gait speed	4 (4-4)	4 (4-4)	0 (0-0)	0	1.000	4 (4-4)	0 (0-0)	1.890	0.059

DASI, Duke activity status index; DFIS, daily fatigue impact scale; ESS, Epworth sleepiness score; HADS, hospital anxiety and depression scale; ISWT, incremental shuttle walking test; SPPB, short physical performance battery.
*Median changes at 6 and 12 weeks calculated by averaging per-participant delta values taken between baseline and the indicated timepoint. Wilcoxon signed rank test was used to evaluate changes from baseline to week 6 and to week 12.

without negatively impacting on their levels of fatigue. Exercising at a moderate intensity has also been shown to impact on communication pathways between the central nervous system and peripheries regarding energy homeostasis. This has a potential positive impact on cognition and has also been linked to improved cellular energy metabolism with a reduction in oxidative stress and could offer a theory as to the observed changes in our study.[59]

The flexibility and supported approach to our exercise programme allowed patients to participate safely within their home environment, without the need for specialist equipment, allowing for greater autonomy. The use of remote monitoring and support in the early phase of the programme (weeks 0-6), provided through weekly telehealth calls, allowed for real time adaptation, problem solving, and the ability to monitor barriers to adherence. Despite withdrawal of telehealth calls at week 6, a reduction in fatigue severity continued to be observed until week 12. Thus, whilst specialist support is likely beneficial initially, participants may become confident in their own level of physical activity with less dependence on health-care professionals over time.

Changes in daytime somnolence and cognitive symptoms are also likely key to reducing fatigue in patients with PBC. One theory is that a combination of better sleep and improved mentation has the potential to improve central fatigue, by addressing abnormalities in cortical inhibitory/excitatory circuits.[33] However, in the absence of objective testing this can only be speculated. Exercise interventions may also influence anxiety and depression, as reported in several chronic diseases.[60,61] Across our cohort, the baseline prevalence of anxiety and depression was high according to the HADS.[62] Whilst we observed an improvement in the HADS over time, scores remained within the 'abnormal' category at 12 weeks. This is perhaps unsurprising, given that targeted psychological support was not provided as part of our programme. Alongside studying exercise, greater understanding of the psychological needs of patients are required for a holistic 'HRQoL-targeted' intervention to be of value.[63] Whilst it is not known by how much the ESS should fall to be considered clinically meaningful, a reduction of >25% and or to below a threshold of 10 have been proposed – both of which were observed following 12 weeks of HBEP.[64,65]

Our study is not without limitations, given the small sample size (hindered by emergence of the COVID-19 pandemic) and lack of a matched control group. By design, the HBEP was evaluated in a pilot feasibility study, and thus there is potential of selection bias. Secondly, individuals with hepatic decompensation and/or persistent jaundice were excluded. This is because as liver disease progresses, the pathogenesis of fatigue becomes more complex, with mechanisms that are not specific to disease aetiology, autoimmunity, or cholestasis. Additionally, even though adherence was subjectively maintained after withdrawal of weekly telehealth calls, the longevity of compliance needs to be explored beyond 12 weeks.

The PBC-40 HRQoL tool, whilst robustly validated in patients with PBC, is also not sensitive enough to discriminate between central and peripheral fatigue. Moreover, in the absence of muscle biopsies, calorimetry or magnetic resonance spectroscopy, the effects of exercise intervention on bioenergetics require further study in dedicated randomised trials. We must also acknowledge the intensity at which

participants completed the HBEP, as this is open to subjective bias. Indeed, our programme relied on individuals self-reporting their perceived efforts, and despite education regarding work rate capacity, it is possible that participants exercised at lower intensities than reported. However, changes in aerobic and functional capacity suggest participants worked at a moderate intensity, but in the absence of close objective monitoring this can only be hypothesised.

It is also important to note that 19% of our study cohort were not being treated with UDCA due to reported intolerance. In part, this may be due to the principal trial centre being a tertiary liver unit, receiving referrals from many hospitals around the country, including of patients who are UDCA intolerant. Nevertheless, the rate of non-UDCA-treated patients is broadly reflective of the UK-PBC population as a whole,[3] and similar to that captured in other countries.[66] It is therefore important for future studies of exercise intervention to be conducted in larger group of patients, along with dedicated sub-group analysis of UDCA-treated *vs.* non-UDCA-treated individuals. Lastly, participants were not blinded to the primary aim of the intervention, which due to significant ethical concerns, and staunch objections from our patient and public involvement group, was not possible. This may have led to patients 'expecting' clinical benefit from study outset, leading to an overestimation of true treatment effect. Indeed, such phenomena have been observed in symptom studies of anti-itch therapy in PBC, wherein patients report subjective clinical benefit despite occupying the placebo arm of a randomised-controlled trial.[53]

Future randomised trials of exercise interventions need to be supported by efficacy and mechanistic evaluations, determining if (and how) HBEP benefits patients. Remote monitoring of physical activity through electronic applications or smart watch technology would also allow clinicians to monitor compliance, rather than relying on subjective patient diaries. As the impact of fatigue differs greatly between individuals, any intervention needs to offer a variety of exercises that could be progressed or regressed while still improving functional/aerobic capacity. Given the intervention phases, a broader choice of exercises may encourage longer term adherence in clinical practice and most importantly optimise patient autonomy.

In conclusion, our study highlights that a novel HBEP, incorporating straightforward low impact body-weighted exercise, is safe and feasible, and may have the potential to attenuate fatigue and daytime somnolence while improving functional capacity in PBC. These early findings may be applied to design future and larger controlled studies, to investigate the effects of HBEP on peripheral and central fatigue, alongside broader domains of HRQoL.

Affiliations

[1]*National Institute of Health Research (NIHR) Birmingham Biomedical Research Centre (BRC), Centre for Liver and Gastrointestinal Research, University of Birmingham, Birmingham, UK;* [2]*Liver Unit, University Hospitals Birmingham Queen Elizabeth. Birmingham UK;* [3]*School of Sport, Exercise and Rehabilitation Sciences, University of Birmingham, Birmingham, UK;* [4]*Institute of Immunology and Immunotherapy, University of Birmingham, Birmingham, UK;* [5]*Institute of Applied Health Research, University of Birmingham, UK*

Abbreviations

ESS, Epworth sleepiness score; HADS, hospital anxiety and depression scale; HBEP, home-based exercise programme; HRQoL, health-related quality of life; PBC, primary biliary cholangitis; RPE, rate of perceived exertion; SPPB, short physical performance battery; UDCA, ursodeoxycholic acid.

Conflicts of interest

The authors of this study declare that they do not have any conflict of interest.

Please refer to the accompanying ICMJE disclosure forms for further details.

Authors' contributions

AF/FW: data extraction, study design, data and statistical analysis, writing of first draft of the manuscript including figures. AF/FW/SD: delivery of study intervention and reviewing/editing manuscript drafts through to submission. JH/PJT/AF: participant identification and reviewing/editing manuscript drafts through to submission. MJA/PJT: study concept and design, data and statistical analysis, revision of manuscript drafts through to submission (and resubmission). PJT: Study conception and guarantor of the article.

Data availability statement

Data is available upon request from the corresponding author.

Financial support

Palak J. Trivedi receives institutional salary support from the National Institute for Health Research (NIHR) Birmingham Biomedical Research Centre (BRC). This paper presents independent research supported by the Birmingham NIHR BRC based at the University Hospitals Birmingham National Health Service (NHS) Foundation Trust and the University of Birmingham. The views expressed are those of the author(s) and not necessarily those of the NHS, the NIHR, or the Department of Health. This paper presents independent research supported by the BRC based at the University Hospitals Birmingham NHS Foundation Trust and the University of Birmingham. This study was supported by an unrestricted grant provided by Intercept Pharmaceuticals.

Supplementary data

Supplementary data to this article can be found online at https://doi.org/10.1016/j.jhepr.2024.101210.

References

Author names in bold designate shared co-first authorship

[1] Lleo A, Wang G-Q, Gershwin ME, Hirschfield GM. Primary biliary cholangitis. The Lancet 2020;396:1915–1926. https://doi.org/10.1016/S0140-6736(20)31607-X.

[2] Trivedi PJ, Hirschfield GM. Recent advances in clinical practice: epidemiology of autoimmune liver diseases. Gut 2021;70:1989–2003. https://doi.org/10.1136/gutjnl-2020-322362.

[3] **Carbone M**, **Mells GF**, **Pells G**, et al. Sex and age are determinants of the clinical phenotype of primary biliary cirrhosis and response to ursodeoxycholic Acid. Gastroenterology 2013;144:560–569.e7. https://doi.org/10.1053/j.gastro.2012.12.005.

[4] Carbone M, Sharp SJ, Flack S, et al. The UK-PBC Risk Scores: derivation and validation of a scoring system for long-term prediction of end-stage liver disease in primary biliary cirrhosis. Hepatology 2015. https://doi.org/10.1002/hep.28017.

[5] Lammers WJ, Hirschfield GM, Corpechot C, et al. Development and validation of a scoring system to predict outcomes of patients with primary biliary cirrhosis receiving ursodeoxycholic acid therapy. Gastroenterology 2015. https://doi.org/10.1053/j.gastro.2015.07.061.

[6] Harms MH, van Buuren HR, Corpechot C, et al. Ursodeoxycholic acid therapy and liver transplant-free survival in patients with primary biliary cholangitis. J Hepatol 2019;71:357–365. https://doi.org/10.1016/j.jhep.2019.04.001.

[7] **Murillo Perez CF**, **Fisher H**, Hiu S, et al. Greater transplant-free survival in patients receiving obeticholic acid for primary biliary cholangitis in a clinical trial setting compared to real-world external controls. Gastroenterology 2022;S0016–5085(22):01060-5. https://doi.org/10.1053/j.gastro.2022.08.054.

[8] **Trivedi PJ**, **Lammers WJ**, van Buuren HR, et al. Stratification of hepatocellular carcinoma risk in primary biliary cirrhosis: a multicentre international study. Gut 2016;65:321–329. https://doi.org/10.1136/gutjnl-2014-308351.

[9] **Gungabissoon U**, **Gibbons DC**, Requena G, et al. Disease burden of primary biliary cholangitis and associated pruritus based on a cross-sectional US claims analysis. BMJ Open Gastroenterol 2022;9:e000857. https://doi.org/10.1136/bmjgast-2021-000857.
[10] Montali L, Gragnano A, Miglioretti M, et al. Quality of life in patients with primary biliary cholangitis: a cross-geographical comparison. J Transl Autoimmun 2021;4:100081. https://doi.org/10.1016/j.jtauto.2021.100081.
[11] Dyson JK, Wilkinson N, Jopson L, et al. The inter-relationship of symptom severity and quality of life in 2055 patients with primary biliary cholangitis. Aliment Pharmacol Ther 2016;44:1039–1050. https://doi.org/10.1111/apt.13794.
[12] **Mells GF**, **Pells G**, Newton JL, et al. Impact of primary biliary cirrhosis on perceived quality of life: the UK-PBC national study. Hepatology 2013;58:273–283. https://doi.org/10.1002/hep.26365.
[13] Jones DE, Al-Rifai A, Frith J, et al. The independent effects of fatigue and UDCA therapy on mortality in primary biliary cirrhosis: results of a 9 year follow-up. J Hepatol 2010;53:911–917. https://doi.org/10.1016/j.jhep.2010.05.026.
[14] Björnsson E, Kalaitzakis E, Neuhauser M, et al. Fatigue measurements in patients with primary biliary cirrhosis and the risk of mortality during follow-up. Liver Int 2010;30:251–258. https://doi.org/10.1111/j.1478-3231.2009.02160.x.
[15] **Khanna A**, **Jopson L**, Howel D, et al. Rituximab is ineffective for treatment of fatigue in primary biliary cholangitis: a phase 2 randomized controlled trial. Hepatology 2018. https://doi.org/10.1002/hep.30099.
[16] **Pells G**, **Mells GF**, **Carbone M**, et al. The impact of liver transplantation on the phenotype of primary biliary cirrhosis patients in the UK-PBC cohort. J Hepatol 2013. https://doi.org/10.1016/j.jhep.2013.02.019.
[17] Carbone M, Bufton S, Monaco A, et al. The effect of liver transplantation on fatigue in patients with primary biliary cirrhosis: a prospective study. J Hepatol 2013;59:490–494. https://doi.org/10.1016/j.jhep.2013.04.017.
[18] Mosher VAL, Swain MG, Pang JXQ, et al. Primary biliary cholangitis alters functional connections of the brain's deep gray matter. Clin Transl Gastroenterol 2017;8:e107. https://doi.org/10.1038/ctg.2017.34.
[19] Mosher VAL, Swain MG, Pang JXQ, et al. Magnetic resonance imaging evidence of hippocampal structural changes in patients with primary biliary cholangitis. Clin Transl Gastroenterol 2018;9:169. https://doi.org/10.1038/s41424-018-0038-z.
[20] Hollingsworth KG, Macgowan GA, Morris L, et al. Cardiac torsion-strain relationships in fatigued primary biliary cirrhosis patients show accelerated aging: a pilot cross-sectional study. J Appl Physiol (1985) 2012;112:2043–2048. https://doi.org/10.1152/japplphysiol.00195.2012.
[21] Newton JL, Hudson M, Tachtatzis P, et al. Population prevalence and symptom associations of autonomic dysfunction in primary biliary cirrhosis. Hepatology 2007;45:1496–1505. https://doi.org/10.1002/hep.21609.
[22] Hollingsworth KG, Newton JL, Robinson L, et al. Loss of capacity to recover from acidosis in repeat exercise is strongly associated with fatigue in primary biliary cirrhosis. J Hepatol 2010;53:155–161. https://doi.org/10.1016/j.jhep.2010.02.022.
[23] Phaw NA, Dyson JK, Mells G, Jones D. Understanding fatigue in primary biliary cholangitis. Dig Dis Sci 2020. https://doi.org/10.1007/s10620-020-06502-0.
[24] **Jones DEJ**, **Hollingsworth K**, Fattakhova G, et al. Impaired cardiovascular function in primary biliary cirrhosis. Am J Physiol Gastrointest Liver Physiol 2010;298:G764–G773. https://doi.org/10.1152/ajpgi.00501.2009.
[25] Newton JL, Elliott C, Frith J, et al. Functional capacity is significantly impaired in primary biliary cirrhosis and is related to orthostatic symptoms. Eur J Gastroenterol Hepatol 2011;23:566–572. https://doi.org/10.1097/MEG.0b013e3283470256.
[26] Huisinga JM, Filipi ML, Stergiou N. Elliptical exercise improves fatigue ratings and quality of life in patients with multiple sclerosis. J Rehabil Res Dev 2011;48:881–890. https://doi.org/10.1682/jrrd.2010.08.0152.
[27] **Langeskov-Christensen M**, **Bisson EJ**, et al. Potential pathophysiological pathways that can explain the positive effects of exercise on fatigue in multiple sclerosis: a scoping review. J Neurol Sci 2017;373:307–320. https://doi.org/10.1016/j.jns.2017.01.002.
[28] Zghal F, Cottin F, Kenoun I, et al. Improved tolerance of peripheral fatigue by the central nervous system after endurance training. Eur J Appl Physiol 2015;115:1401–1415. https://doi.org/10.1007/s00421-015-3123-y.
[29] Puetz TW, Flowers SS, O'Connor PJ. A randomized controlled trial of the effect of aerobic exercise training on feelings of energy and fatigue in sedentary young adults with persistent fatigue. Psychother Psychosom 2008;77:167–174. https://doi.org/10.1159/000116610.
[30] Avaux M, Hoellinger P, Nieuwland-Husson S, et al. Effects of two different exercise programs on chronic fatigue in lupus patients. Acta Clin Belg 2016;71:403–406. https://doi.org/10.1080/17843286.2016.1200824.
[31] Cheville AL, Kollasch J, Vandenberg J, et al. A home-based exercise program to improve function, fatigue, and sleep quality in patients with Stage IV lung and colorectal cancer: a randomized controlled trial. J Pain Symptom Manage 2013;45:811–821. https://doi.org/10.1016/j.jpainsymman.2012.05.006.
[32] **Van Herck M**, **Antons J**, Vercoulen JH, et al. Pulmonary rehabilitation reduces subjective fatigue in COPD: a responder analysis. J Clin Med 2019;8:1264. https://doi.org/10.3390/jcm8081264.
[33] McDonald C, Newton J, Lai HM, et al. Central nervous system dysfunction in primary biliary cirrhosis and its relationship to symptoms. J Hepatol 2010;53:1095–1100. https://doi.org/10.1016/j.jhep.2010.05.036.
[34] Scherr J, Wolfarth B, Christle JW, et al. Associations between Borg's rating of perceived exertion and physiological measures of exercise intensity. Eur J Appl Physiol 2013;113:147–155. https://doi.org/10.1007/s00421-012-2421-x.
[35] **Freer A**, **Williams F**, Durman S, et al. Home-based exercise in patients with refractory fatigue associated with primary biliary cholangitis: a protocol for the EXerCise Intervention in cholesTatic LivEr Disease (EXCITED) feasibility trial. BMJ Open Gastroenterol 2021;8:e000579. https://doi.org/10.1136/bmjgast-2020-000579.
[36] **Jacoby A**, **Rannard A**, Buck D, et al. Development, validation, and evaluation of the PBC-40, a disease specific health related quality of life measure for primary biliary cirrhosis. Gut 2005;54:1622–1629. https://doi.org/10.1136/gut.2005.065862.
[37] Singh SJ, Morgan MD, Scott S, et al. Development of a shuttle walking test of disability in patients with chronic airways obstruction. Thorax 1992;47:1019–1024. https://doi.org/10.1136/thx.47.12.1019.
[38] Hlatky MA, Boineau RE, Higginbotham MB, et al. A brief self-administered questionnaire to determine functional capacity (the Duke Activity Status Index). Am J Cardiol 1989;64:651–654. https://doi.org/10.1016/0002-9149(89)90496-7.
[39] Ronai P, Gallo PM. The short physical performance battery (ASSESSMENT). ACSM's Health Fitness J 2019;23:52–56. https://doi.org/10.1249/FIT.0000000000000519.
[40] Williams FR, Vallance A, Faulkner T, et al. Home-based exercise therapy in patients awaiting liver transplantation: protocol for an observational feasibility trial. BMJ Open 2018;8. https://doi.org/10.1136/bmjopen-2017-019298.
[41] Bager P, Bossen L, Gantzel R, Grønbæk H. High-dose oral thiamine versus placebo for chronic fatigue in patients with primary biliary cholangitis: a crossover randomized clinical trial. PLoS One 2024;19:e0301354. https://doi.org/10.1371/journal.pone.0301354.
[42] Frith J, Newton J. Fatigue impact scale. Occup Med (Lond) 2010;60:159. https://doi.org/10.1093/occmed/kqp180.
[43] Johns MW. A new method for measuring daytime sleepiness: the Epworth sleepiness scale. Sleep 1991;14:540–545. https://doi.org/10.1093/sleep/14.6.540.
[44] Snaith RP. The hospital anxiety and depression scale. Health Qual Life Outcomes 2003;1:29. https://doi.org/10.1186/1477-7525-1-29.
[45] Younossi ZM, Guyatt G, Kiwi M, et al. Development of a disease specific questionnaire to measure health related quality of life in patients with chronic liver disease. Gut 1999;45:295–300. https://doi.org/10.1136/gut.45.2.295.
[46] Corpechot C, Chazouillères O, Poupon R. Early primary biliary cirrhosis: biochemical response to treatment and prediction of long-term outcome. J Hepatol 2011;55:1361–1367. https://doi.org/10.1016/j.jhep.2011.02.031.
[47] I Rigopoulou E, Bakarozi M, Dimas I, et al. Total and individual PBC-40 scores are reliable for the assessment of health-related quality of life in Greek patients with primary biliary cholangitis. J Transl Int Med 2023;11:246–254. https://doi.org/10.2478/jtim-2023-0098.
[48] Corpechot C, Chazouillères O, Rousseau A, et al. A placebo-controlled trial of bezafibrate in primary biliary cholangitis. N Engl J Med 2018;378:2171–2181. https://doi.org/10.1056/NEJMoa1714519.
[49] Tanaka A, Hirohara J, Nakano T, et al. Association of bezafibrate with transplant-free survival in patients with primary biliary cholangitis. J Hepatol 2021;75:565–571. https://doi.org/10.1016/j.jhep.2021.04.010.
[50] Nevens F, Andreone P, Mazzella G, et al. A placebo-controlled trial of obeticholic acid in primary biliary cholangitis. N Engl J Med 2016;375:631–643. https://doi.org/10.1056/NEJMoa1509840.
[51] Hirschfield GM, Bowlus CL, Mayo MJ, et al. A phase 3 trial of seladelpar in primary biliary cholangitis. N Engl J Med 2024;390:783–794. https://doi.org/10.1056/NEJMoa2312100.

[52] Kowdley KV, Bowlus CL, Levy C, et al. Efficacy and safety of elafibranor in primary biliary cholangitis. N Engl J Med 2024;390:795–805. https://doi.org/10.1056/NEJMoa2306185.
[53] **Levy C**, **Kendrick S**, Bowlus CL, et al. GLIMMER: a randomized phase 2b dose-ranging trial of linerixibat in primary biliary cholangitis patients with pruritus. Clin Gastroenterol Hepatol 2023;21:1902–1912.e13. https://doi.org/10.1016/j.cgh.2022.10.032.
[54] **Zenouzi R**, **von der Gablentz J**, Heldmann M, et al. Patients with primary biliary cholangitis and fatigue present with depressive symptoms and selected cognitive deficits, but with normal attention performance and brain structure. PLoS One 2018;13:e0190005. https://doi.org/10.1371/journal.pone.0190005.
[55] **Abbas N**, **Smith R**, Flack S, et al. Critical shortfalls in the management of PBC: results of a UK-wide, population-based evaluation of care delivery. JHEP Rep 2024;6:100931. https://doi.org/10.1016/j.jhepr.2023.100931.
[56] Rasmussen MK, Juel C, Nordsborg NB. Exercise-induced regulation of muscular Na+-K+ pump, FXYD1, and NHE1 mRNA and protein expression: importance of training status, intensity, and muscle type. Am J Physiol Regul Integr Comp Physiol 2011;300:R1209–R1220. https://doi.org/10.1152/ajpregu.00635.2010.
[57] Juel C. Muscle pH regulation: role of training. Acta Physiol Scand 1998;162:359–366. https://doi.org/10.1046/j.1365-201X.1998.0305f.x.
[58] Dela F, Holten M, Juel C. Effect of resistance training on Na,K pump and Na+/H+ exchange protein densities in muscle from control and patients with type 2 diabetes. Pflugers Arch 2004;447:928–933. https://doi.org/10.1007/s00424-003-1213-x.
[59] Voss MW, Carr LJ, Clark R, Weng T. Revenge of the “sit” II: does lifestyle impact neuronal and cognitive health through distinct mechanisms associated with sedentary behavior and physical activity? Ment Health Phys Activity 2014;7:9–24. https://doi.org/10.1016/j.mhpa.2014.01.001.
[60] Carli F, Scheede-Bergdahl C. Prehabilitation to enhance perioperative care. Anesthesiol Clin 2015;33:17–33. https://doi.org/10.1016/j.anclin.2014.11.002.
[61] Fibbins H, Lederman O, Rosenbaum S. Chapter 21 - get moving: physical activity and exercise for mental health. In: Badcock JC, Paulik G, editors. A clinical introduction to psychosis. Academic Press; 2020. p. 493–510. https://doi.org/10.1016/B978-0-12-815012-2.00021-3.
[62] Crawford JR, Henry JD, Crombie C, Taylor EP. Normative data for the HADS from a large non-clinical sample. Br J Clin Psychol 2001;40:429–434. https://doi.org/10.1348/014466501163904.
[63] Watt M, Hyde A, Johnson E, et al. An online mind-body program improves mental health and quality of life in primary biliary cholangitis: a randomized controlled trial. Hepatol Commun 2023;7:e0316. https://doi.org/10.1097/HC9.0000000000000316.
[64] Newton JL, Gibson GJ, Tomlinson M, et al. Fatigue in primary biliary cirrhosis is associated with excessive daytime somnolence. Hepatology 2006;44:91–98. https://doi.org/10.1002/hep.21230.
[65] Jones DEJ, Newton JL. An open study of modafinil for the treatment of daytime somnolence and fatigue in primary biliary cirrhosis. Aliment Pharmacol Ther 2007;25:471–476. https://doi.org/10.1111/j.1365-2036.2006.03223.x.
[66] Marschall H-U, Henriksson I, Lindberg S, et al. Incidence, prevalence, and outcome of primary biliary cholangitis in a nationwide Swedish population-based cohort. Sci Rep 2019;9:11525. https://doi.org/10.1038/s41598-019-47890-2.

Keywords: Autoimmune Liver Disease; Cholestasis; Functional Capacity; Health Related Quality of Life; Patient Reported Outcome Measure; Symptoms.
Received 13 May 2024; received in revised form 24 August 2024; accepted 29 August 2024; Available online 6 September 2024

Journal of Hepatology, Volume *6*

Supplemental information

A home-based exercise programme attenuates fatigue in primary biliary cholangitis: Results from the EXCITED clinical trial

Alice Freer, Felicity R. Williams, Simon Durman, Jennifer Hayden, Matthew J. Armstrong, and Palak J. Trivedi

A home-based exercise programme attenuates fatigue in primary biliary cholangitis: Results from the EXCITED clinical trial

Alice Freer, Felicity R Williams, Simon Durman, Jennifer Hayden, Matthew J Armstrong, Palak J. Trivedi

Table of contents

Supplementary file 1……………………………………………………………………2

Fig. S1……………………………………………………………………………………8

Fig. S2……………………………………………………………………………………9

Fig. S3…………………………………………………………………………………..10

Table S1………………………………………………………………………………...11

Table S2………………………………………………………………………………...12

HRA and HCRW approval letter……………………………………………………...13

UHB approval letter……………………………………………………………………20

Participant information sheet…………………………………………………………36

BMJ Open Gastroenterology

Home-based exercise in patients with refractory fatigue associated with primary biliary cholangitis: a protocol for the EXerCise Intervention in cholesTatic LivEr Disease (EXCITED) feasibility trial

Alice Freer,[1,2] Felicity Williams,[1,3] Simon Durman,[4] Jennifer Hayden,[2] Palak J Trivedi ,[1,2,5] Matthew J Armstrong[2]

To cite: Freer A, Williams F, Durman S, *et al*. Home-based exercise in patients with refractory fatigue associated with primary biliary cholangitis: a protocol for the EXerCise Intervention in cholesTatic LivEr Disease (EXCITED) feasibility trial. *BMJ Open Gastro* 2021;**8**:e000579. doi:10.1136/bmjgast-2020-000579

► Additional material is published online only. To view please visit the journal online (http://dx.doi.org/10.1136/bmjgast-2020-000579).

PJT and MJA contributed equally.

Received 23 November 2020
Revised 19 January 2021
Accepted 24 January 2021

Check for updates

© Author(s) (or their employer(s)) 2021. Re-use permitted under CC BY-NC. No commercial re-use. See rights and permissions. Published by BMJ.

For numbered affiliations see end of article.

Correspondence to
Dr Palak J Trivedi;
p.j.trivedi@bham.ac.uk

ABSTRACT

Introduction Fatigue is the most commonly reported symptom of the liver disease primary biliary cholangitis (PBC). It affects 40%–80% of patients, has no effective treatment and is associated with heightened mortality risk. The pathogenesis is unknown, but muscle bioenergetic abnormalities have been proposed to contribute. Directly observed exercise has been shown to attenuate symptoms in small groups; however, due to the rare nature of the disease, home-based interventions need to be evaluated for feasibility, safety and efficacy.

Methods and analysis This is a phase 1/pilot, single-arm, open-label clinical trial evaluating a novel home-based exercise programme in patients with PBC with severe fatigue. Forty patients with moderate-severe fatigue (PBC40 fatigue domain score >33; other causes of fatigue excluded) will be selected using a convenience sampling method. A 12-week home-based exercise programme, consisting of individualised resistance, aerobic exercises and telephone health calls (first 6 weeks only), will be delivered. Measures of fatigue (PBC40 fatigue domain; fatigue impact scale), quality of life, sleep (Epworth Sleep Score), physical activity, anxiety and depression, aerobic exercise capacity (incremental shuttle walk test; Duke Activity Status Index) and functional capacity (short physical performance battery) will be assessed at baseline and at 6 and 12 weeks following the intervention.

Ethics and dissemination The protocol is approved by the National Research Ethics Service Committee London (IRAS 253115). Recruitment commenced in April 2019 and ended in March 2020. Participant follow-up is due to finish by December 2020. Findings will be disseminated through peer-reviewed publication, conference presentation and social media.

Trial registration number NCT04265235.

INTRODUCTION

Primary biliary cholangitis (PBC) is a chronic, immune-mediated liver disease characterised by progressive reduction in bile flow (cholestasis) that leads to biliary fibrosis and eventual cirrhosis.[1] The exact disease mechanisms are unknown but considered multifactorial, encompassing inherited traits and ill-defined environmental factors.[2] Incident rates suggest c35 per 100 000 individuals are affected with PBC, of whom 90% are women aged between 30 and 65 years at the time of diagnosis.[3]

Debilitating, refractory fatigue is one of the most commonly reported symptoms, affecting between 40% and 80% of patients.[4] Although not an indicator of liver disease severity, fatigue is pronounced among individuals of young presenting age and is associated with worse liver transplant-free survival, reduced physical activity and poorer quality of life (QoL) for patients. While a consensus biological explanation is lacking, preclinical data have identified central and peripheral components, alongside suppressed anaerobic threshold during physical exertion.[5 6] Notably, patients with PBC suffering from fatigue report that it is not a single episode of activity they find difficult, rather the incapacity to perform exercise over a sustained period of time. This relates to excessive acidosis building up in peripheral muscle coupled with delayed pH recovery, which tends to manifest after only a minimal duration of physical exertion, compared with non-fatigued patients with PBC and healthy control subjects.[5 7] Importantly, this pathology can be reversed through repeated single exercise episodes, suggesting that the capacity to improve muscle bioenergetics

is retained.[5] Most strikingly, supervised graded exercise intervention demonstrates significant and quantifiable improvements in fatigue severity, together with symptom reduction in domains related to emotional and social dysfunction.[5]

While patients with liver disease report positive attitudes towards the benefits of supervised exercise, they admit to lack of confidence were they to initiate such activity themselves.[5 8] Moreover, those living with fatigue have a natural inclination towards decreased levels of physical activity in response to symptoms.[9] Similar to other chronic diseases, individuals are more likely to disengage, become socially isolated and increase sedentary living due to fear of exacerbating fatigue if correct guidance is not provided.[10] The resulting physical inactivity is a catalyst for muscular degeneration, with reduced exercise tolerance and functional capacity, thereby increasing fatigability.[9 10] This 'fatigability' further exacerbates fear and sedentary living, leading to a vicious cycle of debilitating decline.[9] The consequence of this is further physical inactivity and muscular degeneration, a reduction in oxygen-handling (oxygen consumption peak) and functional capacity, and subsequently the speed at which the muscle fatigues.[9–11]

Access to healthcare professionals appears to reduce self-reported fatigue and positively influence the attitudes towards exercise and the long-term benefits on overall QoL.[12–14] Moreover, home-based exercise programmes (HBEPs) performed at moderate intensity (rate of perceived exertion (RPE) 12–14) have been shown to be safe and improve functional capacity in patients with end-stage liver disease.[15] However, there are no studies that explore the feasibility and efficacy of a moderate-intensity HBEP on improving fatigue or functional capacity in patients with PBC.

Prior to performing a dedicated randomised controlled trial (RCT), a pilot feasibility study is required to determine whether intervention can be conducted within patients' homes, determine whether a larger programme is possible and highlight preferential design features. To this effect, our aim is to conduct a single-centre trial of a structured HBEP, which attenuates fatigue associated with PBC. Efficacy assessment will be through validated health-related quality of life (HRQoL) measures, and fatigue-specific and functionality assessment tools.

METHODS AND ANALYSIS

Study design overview

This is a single-arm, single-centre feasibility trial of a graded HBEP for the treatment of chronic fatigue in patients with PBC. Patients will be recruited through the Liver Unit based at the University Hospitals Birmingham (UHB) National Health Service (NHS) Foundation Trust, UK. An individualised, graded HBEP will be delivered for 12 weeks to investigate safety, adherence and treatment efficacy (figure 1). Fatigue, HRQoL, functional capacity and sleep quality will be recorded at baseline and at 6 and 12 weeks following initial assessment.

Sample and selection

Patients attending the dedicated PBC clinic will be presented with a PBC40 questionnaire, which is a robustly validated HRQoL measure specifically for application in PBC.[16] Given that some patients experience fatigue by virtue of having advanced disease, our study will exclusively target individuals who have clinically significant symptoms although with fully compensated liver function. Patients with clinically significant fatigue (defined by a PBC40 fatigue domain score >33) will be asked to complete a second questionnaire remotely, 14 days after initial assessment.[16 17] Patients with persistently elevated scores (in the absence of other contributory causes) will

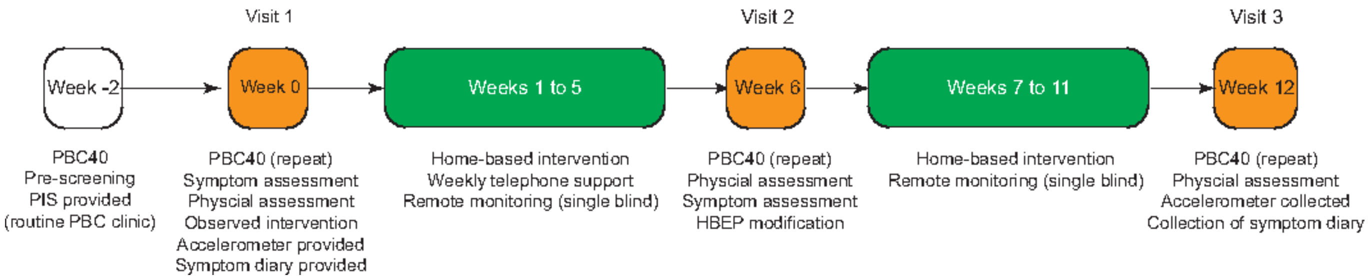

Figure 1 Study overview. Patients with moderate-severe fatigue (PBC40 fatigue domain score >33) will be identified from clinic and PBC40 fatigue assessment completed. Eligible participants will be invited to attend a dedicated screening visit within 2 weeks and a repeat PBC40 questionnaire completed. After obtaining consent, the investigator will perform full physical and symptom assessment of the trial participant and demonstrate the intended intervention. The trial participant will then be observed while performing the aforementioned intervention and will be provided a symptom diary and single-blinded GeneActiv accelerometer for home use (daily activity monitor readings will be captured remotely by the investigator, but not visible by the participant). Thereafter, the participant will be instructed to perform a tailored, daily HBEP by the investigators (liver physiotherapist and personal trainer). Weekly telephone support will be provided in the first 6 weeks (interval between visit 1 and visit 2), together with modifications to the exercise programme as needed. At week 6, the trial participant will be invited for an interim assessment, followed by another 6 weeks of intervention. Weekly telephone support will be withdrawn between weeks 6 and 12 (end of the study). Assessment of the primary efficacy measure will be performed at week 12 (end of study visit). HBEP, home-based exercise programme; PBC, primary biliary cholangitis; PIS, patient information sheet.

be deemed eligible for trial participation. Additional criteria for trial entry include the following.

Inclusion criteria

- ≥ 18 years old.
- A confirmed diagnosis of PBC, in keeping with international guidelines.[18]

Exclusion criteria

- Decompensated liver disease, as evident by the presence of ascites, hepatic encephalopathy (any grade) and history of variceal bleeding.
- Total bilirubin of >50 µmol/L, in the absence of Gilbert's syndrome.
- Prior liver transplantation.
- On the waitlist for or likely to need referral for liver transplantation in the next 6 months (judged by the principal investigator (PI)).
- Refractory pruritus (judged by the PI).
- No dual-energy X-ray absorptiometry scan within 5 years.
- Untreated osteoporosis.
- Cardiovascular instability (judged by PI).
- Untreated hypovitaminosis or anaemia.
- Untreated hypothyroidism.
- Untreated coeliac disease.
- WHO performance status ≥3.
- History of unexplained falls.
- Neither patient nor next of kin are English speaking.
- Refusal or lack of capacity to give informed consent.
- Participation in another interventional trial for PBC in the last 3 months.
- Concomitant excess alcohol consumption (>14 units for women; >21 for men).
- Concomitant liver disease of another aetiology, including (but not limited to) viral hepatitis, autoimmune hepatitis, primary sclerosing cholangitis, alcohol-induced liver disease, non-alcoholic fatty liver disease, Wilson disease, alpha-1-antitrypsin deficiency, Budd-Chiari syndrome, hereditary haemochromatosis, drug-induced liver injury and IgG4-related disease.
- Intercurrent active or latent infection.
- Intercurrent immunocompromised state.
- Concurrent chemotherapy.
- Active malignancy (other than non-melanomatous skin cancer).

Once screened and deemed eligible, patients will be contacted via telephone to discuss their involvement within the study, and a patient information sheet will sent sent by the chief investigator (CI) or co-investigator (Co-I). If individuals agree to take part, an appointment will be arranged for written consent, baseline assessment and an initial supervised exercise session.

Study visits

Patients who suffer from PBC attend regular clinic appointments as part of routine clinical care. Where possible, all study visits will be conducted on the same day as routine clinic appointments to reduce the burden of hospital attendance and travel.

The study requires three separate attendances to the clinic: at week 0 (visit 1—baseline), week 6 (visit 2) and week 12 (visit 3—end of study) (figure 1). At each visit, the assessment of fatigue, HRQoL, functional capacity and sleep quality will be quantified.

Visit 1

At week 0 (baseline), fatigue will be quantified using the PBC40 fatigue domain and Fatigue Impact Scale (FIS).[19] Additional metrics recorded at baseline will include the following:

- Remaining components of the PBC40 assessment tool.[16]
- The Chronic Liver Disease Questionnaire (CLDQ).[20]
- The Hospital Anxiety and Depression Scale (HADS)[21]
- The Epworth Sleep Score (ESS).[22]
- Measures of functional and aerobic capacity:
  - Incremental shuttle walking test (ISWT).[23 24]
  - Short performance physical battery (SPPB).[25]
  - Duke Activity Status Index (DASI).[26]

Baseline assessment will also capture anthropometric data (age, sex, weight, height and body mass index), details of PBC-specific treatments (including ursodeoxycholic acid (UDCA), obeticholic acid, fibric acid derivatives, bile acid sequestrants, rifampicin, selective serotonin reuptake antagonists, opioid antagonists), dietetic input (eg, use of protein supplements) and laboratory data (liver biochemistry, renal function, thyroid function, vitamin D levels, haemoglobin A1c, full blood count and clotting profile).

Intervention and monitoring

Participants will be provided with a moderate-intensity, low-impact individualised HBEP dependent on index measures of physical activity, performance status and confidence with intervention during visit 1. Participants will be advised to exercise within a Borg RPE of 12–14, as this has been shown to correlate with anaerobic thresholds in healthy individuals and improve functional capacity in patients with end-stage liver disease.[15 27] In addition, each participant will be given a GENEActiv wrist accelerometer in a single-blind manner (which will provide retrospective feedback on activity to the investigators but not the participants) to wear 24 hours a day during the 12-week study period. The GENEActiv will encompass assessment of physical activity and sleep quality. In parallel, a paper-based patient diary will be provided to record HBEP targets, alongside a copy of their specific programme.[15]

Home-based exercise program

Following discussions with our patient and public involvement group, it was recommended that all interventions should include functional movements, require no specialist equipment, that exercise instructions should be supported with written information and the overall

Table 1 Exercise levels prescribed for participants

Graded level	Exercises	Work phase	Rest phase	Number of sets per exercise
Low	Sit to stand<br>Bench press<br>Frog squat<br>Bear crawl	20 s	40 s	3–5
Low	Sit to stand<br>Bench press<br>Frog squat<br>Bear crawl	30 s	30 s	3–5
Moderate	Sit to stand<br>Frog squat<br>Bear crawl<br>Kick sit<br>Body drop	30 s	30 s	4–5
Moderate	Frog squat<br>Bear crawl<br>Kick sit<br>Body drop<br>Cobra	30 s	30 s	4–5
High	Frog squat<br>Bear crawl<br>Kick sit<br>Body drops<br>Cobra	40 s	20 s	5

commitment should not involve more than three sessions per week. Therefore, at initial assessment, participants will be taught functional resistance exercises that they can complete at home (table 1 and online supplemental appendix 1)—as previously described.[15] Entry levels will be determined depending on individual physical capacity and confidence. Each participant will be observed completing their specific exercise programme during visit 1, under the supervision of an accredited specialist physiotherapist and a qualified personal trainer. Patient technique, safety and confidence in completing each exercise will be assessed, as well as ensuring they can maintain an RPE of 12–14 throughout the exercise session. Participants will be advised to complete functional resistance exercises 2–3 times per week (depending on capability) as this has been shown to correlate with physiological change and will be provided with written information for each exercise to guide completion at home. Although patients will not be directly supervised while conducting exercises at home, remote monitoring through assessment of accelerometer data and weekly telephone calls will be conducted during this time.[15]

The RPE is a psychophysical tool that is widely used to assess the perception of effort during exercise across a multitude of health conditions and positively correlates with both heart rate and blood lactate.[27] Given its simplicity, acceptance within healthcare and assurance that it correlates with physiological change, the use of RPE during execution of the field test and HBEP was deemed an appropriate choice. This will also allow for an individualised approach and inclusivity of participants despite baseline functional ability. Supplementary telephone calls will allow for remote progression and regression of exercises and monitoring of adverse symptoms including exertion-related chest discomfort, light headedness or dizziness.

At the week 6 assessment, the programme will be reviewed and amended (where appropriate) according to participants' functional/aerobic capacity and confidence (table 1 and online supplemental appendix 1).

Between visit 1 and visit 2

Weekly targets and progressions or regressions will be facilitated by supplementary telephone calls from week 0 to week 6.[15] These will be delivered by the physiotherapist to provide remote monitoring and support regarding the HBEP. The focus will be to:

- Assess compliance to the study intervention.
- Discuss the severity of fatigue.
- Discuss adverse effects that have not been reported in the interval prior to last communication with the patient.
- Review of exercises to give patients an opportunity to discuss any concerns or ask questions related to the exercise programme.
- Determine the need for progression or regression of the HBEP, including any individualised modifications that need to be made. For instance, modifications will be made if participants find a particular exercise too difficult (or easy) or are not reaching training zones of a self-reported RPE of 12–14. The activity time can also be increased and rest phase decreased, in the event participants report the programme to not be challenging enough (and vice versa).

Following the week 6 assessment (visit 2), telephone support will be withdrawn to assess long-term adherence to the study intervention.

Visit 2

At week 6 after the intervention, participants will be invited back to re-evaluate all baseline functional/aerobic capacity tests, HRQoL scores, and symptom severity and ensure appropriate adjustments to the HBEP are made.

Visit 3

The intervention will conclude at week 12, at which point investigators will assess all aforementioned parameters a final time. Participant diaries and accelerometers will be collected at this point.

Project objectives

- Determine whether HBEP intervention is feasible and safe.
- Identify whether HBEP attenuates fatigue in patients with PBC.
- Outline the longevity in improvement in symptoms of fatigue and HRQoL once health-related telephone support is withdrawn.

Outcome measures

The overarching goal of this study is to determine the feasibility of HBEP among patients with PBC with clinically significant fatigue, with the decision to proceed to a subsequent RCT made on the following criteria:

- Sample size: >25% of sample population (ie, patients with PBC and fatigue) eligible for study.
- Patient consent: >66% of eligible study population consented to the study.
- Safety: No severe adverse effects related to the study intervention.
- Adherence: >80% adherence with HBEP for a minimum of 6 weeks (measured by self-reported diaries).

Our primary efficacy measure will be a reduction in median fatigue severity score according to the PBC40 QoL assessment tool. The PBC40 HRQoL assessment tool has been developed and extensively validated for use among patients with PBC, with the fatigue domain being the most commonly applied measure of treatment efficacy used in clinical trials.[16–18]

Secondary measures of efficacy will be gauged by changes in the following assessment tools, and justification for use can be found in online supplemental appendix 2:

- FIS.[19]
- The other domains of the PBC40 QoL tool, relating to cognitive, social, emotional, pruritus and overall symptoms.[16]
- ISWT.[23]
- SPPB.[25]
- CLDQ.[20]
- ESS.[22]
- HADS.[21]
- DASI.[26]
- Liver biochemical parameters, namely, serum alkaline phosphatase, bilirubin and alanine transaminase.
- GENEActiv physical activity and sleep quality.

Additional exploratory analysis will be conducted to study associations between fatigue and baseline patient demographics, seasonality and underlying UDCA response status, alongside the other domains of the PBC40 questionnaire.

Data capture

All qualitative and quantitative data will be entered into a purposely designed, secure access database and data subsequently analysed (SPSS V.24; IBM). Feasibility decision rules and clinical outcome measures will be presented using descriptive statistics. Quantitative changes before and after the intervention (from baseline to week 6 and to week 12) will be tested using a non-parametric statistical hypothesis for repeated measurements (Wilcoxon signed-rank test).

Adverse events

There will be a strict process for reporting adverse events (AEs) and adverse reactions (ARs), which will commence at screening and continue until the final participant has completed study intervention. All AEs, serious AEs (SAEs), ARs and serious unexpected ARs will be reported to the CI and sponsor's research and development department via a dedicated case report form (CRF). Only AEs or SAEs that can be deemed probable or absolutely related to the study will be reported to the Research Ethics Committee.

Storage of data

All data relating to the study will be collected by the Co-I and recorded in standardised CRFs. Each participant will be given a unique study number at the time of consent and used as a way of identification. Data will be collected from the participant at point of entry into the study until completion of the intervention. All clinical data will be stored securely as per NHS regulations for a minimum of 15 years. All data documented on the CRF will be entered into an NHS password-secured computer and in concordance with the Data Protection Act 1998. All essential written documentation will be stored in line with the appropriate regulatory requirements and restricted to researchers essential to the study. Coded research data will be stored for 5 years anonymously under the property of the Queen Elizabeth Hospital UHB in keeping with good clinical practice.

Case report forms

CRFs will be completed at week 0 (baseline), week 6 and week 12 (end of study), and include all questionnaire and functional outcomes. Electronic CRFs will be completed on an NHS Trust password protected system and will include medical history, eligibility screening and study treatment adherence.

Sponsorship, indemnity and monitoring

UHB will act as sponsor for the duration of the study. As sponsor, UHB will be responsible for the conduct of the study and indemnify the study centre against any claims, arising from any negligent act or omission by the hospital in fulfilling the sponsor role in respect to the study. The study is supported by an unrestricted grant from Intercept Pharmaceuticals

DISCUSSION

This is the first feasibility trial investigating HBEP in the treatment of refractory fatigue and QoL in patients with PBC. To date, 262 individuals with PBC have been preidentified, of whom 82 are deemed eligible, and 42 agreed to attend a dedicated screening visit.

Safety

Supervised exercise sessions and advice are well supported within chronic disease, but there are very few that focus on home-based exercise and how this influences fatigue. Moreover, there are a limited number of large RCTs that report safe use of exercise therapy in patients with chronic liver injury, most often among patient groups with advanced disease.

Combined with education and the chosen objective measures, participants will have clear training guidelines and supporting information along with contact numbers to reduce the risk of AEs. With supplementary weekly telephone calls across the first 6 weeks, the opportunity to discuss any concerns or AEs will be available.[26] Rigorous exclusion criteria will help to reduce the risk of AEs and maximise the safety of remote exercise intervention that is monitored via health telephone calls. This will allow for maximal participation while ensuring a safe exercise regimen is followed.

The intervention itself has been developed and based on well-documented moderate-intensity training models and extrapolated from an exercise protocol, which demonstrated the safety of such a HBEP in patients with more advanced liver injury.[15]

Challenges to study design

One in 5000 individuals are affected by PBC, with >90% of those being women aged between 30 and 65 years.[3] Consequently, many eligible participants are in some form of employment and have family responsibilities. This will need to be considered when recruiting to the study, and where possible, ensure that research-related visits link with clinic visits to reduce participant burden.

As the study intervention is predominantly home-based, education and support to aid adherence are fundamental. Although it is well recorded that debilitating fatigue and its impact on HRQoL is the most widely reported symptom of PBC, very little is understood about individual influences. To aid in the assessment of adherence, participants will be asked to complete a study diary, with the aim of tracking self-reported physical activity and providing aggregate data in relation to weekly targets. Participants will also receive written information and pictures of their individualised exercise programme, with an opportunity to record exercises digitally. The intervention itself has been developed and based on well-characterised moderate-intensity training models and extrapolated from an exercise protocol that demonstrated the safety of HBEP among patients with more advanced liver injury.[15]

As fatigue differs greatly between individuals, the study intervention needs to offer a variety of exercises that can be progressed and regressed while still improving functional/aerobic capacity. Multiple intensity levels offer a unique approach as have been designed to help ensure the interventions were individually targeted with the flexibility to adapt if needed.

Future RCT considerations

This phase 1, single-centred feasibility study is required not only to demonstrate the safety of the intervention but also to highlight recruitment, dropout rates and adherence. Without this information, it would not be possible to power the correct number of participants needed for future RCTs.

Summary

To the best of our knowledge this is the first study to investigate HBEP in patients with PBC with debilitating fatigue. Enrolment for participation in the study is completed, and final results are expected in early 2021.

Author affiliations
[1]NIHR Birmingham BRC and Centre for Liver and Gastrointestinal Research, University of Birmingham, Birmingham, UK
[2]Liver Unit, University Hospitals Birmingham NHS Foundation Trust, Birmingham, UK
[3]Institute of Immunology and Ageing, University of Birmingham, Birmingham, UK
[4]Department of Physiotherapy, University Hospitals Birmingham NHS Foundation Trust, Birmingham, UK
[5]Centre for Liver and Gastrointestinal Research, University of Birmingham, Birmingham, UK

Twitter Alice Freer @alicefreer18, Felicity Williams @liverphysio and Palak J Trivedi @cholestasisdoc

Acknowledgements We would like to thank the PBC Foundation, PSC Support, and the Birmingham Gastroenterology and Liver Patient and Public Involvement Group for their input and advice into designing the study protocol.

Contributors AF, lead physiotherapist delivering intervention, wrote the first version of the manuscript and approved the final/revised version to submission. FW and SD developed the study protocol design, provided critical insight into manuscript content and approved the final/revised version to submission. JH helped with the study design and selection of eligibility criteria, provided critical insight into manuscript content and approved the final version to submission. PT and MA helped with the study concept and overall supervision of the project and provided critical insight into manuscript content, revisions and final manuscript approval. PT and MA are guarantors of the article.

Funding This work was supported by an unrestricted grant from Intercept Pharmaceuticals.

Competing interests None declared.

Patient consent for publication Not required.

Ethics approval The National Research Ethics Service (NRES) Committee and Health Research Authority London—Riverside Research Ethics Committee (IRAS 253115) approved version 1.1 of the study protocol. All participants will provide informed written consent.

Provenance and peer review Not commissioned; externally peer reviewed.

Data availability statement Data sharing is not applicable as no data sets are generated and/or analysed for this study. Data are available upon reasonable request. All data relevant to the study are included in the article or uploaded as supplementary information. Data sharing plan with regard to study findings will be submitted alongside full trial results.

Supplemental material This content has been supplied by the author(s). It has not been vetted by BMJ Publishing Group Limited (BMJ) and may not have been peer-reviewed. Any opinions or recommendations discussed are solely those of the author(s) and are not endorsed by BMJ. BMJ disclaims all liability and responsibility arising from any reliance placed on the content. Where the content includes any translated material, BMJ does not warrant the accuracy and reliability of the translations (including but not limited to local regulations, clinical guidelines, terminology, drug names and drug dosages), and is not responsible for any error and/or omissions arising from translation and adaptation or otherwise.

Open access This is an open access article distributed in accordance with the Creative Commons Attribution Non Commercial (CC BY-NC 4.0) license, which permits others to distribute, remix, adapt, build upon this work non-commercially, and license their derivative works on different terms, provided the original work is properly cited, appropriate credit is given, any changes made indicated, and the use is non-commercial. See: http://creativecommons.org/licenses/by-nc/4.0/.

ORCID iD
Palak J Trivedi http://orcid.org/0000-0002-4009-8087

REFERENCES

1 Trivedi PJ, Hirschfield GM. Primary biliary cholangitis (formerly primary biliary cirrhosis). *Evidence-based Gastroenterology and Hepatology* 2019:574–91.
2 Trivedi PJ, Cullen S. Etiopathogenesis of primary biliary cirrhosis: an overview of recent developments. *Hepatol Int* 2013;7:28–47.
3 Boonstra K, Beuers U, Ponsioen CY. Epidemiology of primary sclerosing cholangitis and primary biliary cirrhosis: a systematic review. *J Hepatol* 2012;56:1181–8.
4 Carbone M, Mells GF, Pells G, *et al*. Sex and age are determinants of the clinical phenotype of primary biliary cirrhosis and response to ursodeoxycholic acid. *Gastroenterology* 2013;144:560–9.
5 Hollingsworth KG, Newton JL, Robinson L, *et al*. Loss of capacity to recover from acidosis in repeat exercise is strongly associated with fatigue in primary biliary cirrhosis. *J Hepatol* 2010;53:155–61.
6 Phaw NA, Dyson J, Mells G, *et al*. Understanding of central and peripheral fatigue in primary biliary cholangitis. *J Hepatol* 2020;73:S481–2.
7 Goldblatt J, James OF, Jones DE. Grip strength and subjective fatigue in patients with primary biliary cirrhosis. *JAMA* 2001;285:2196–7.
8 Frith J, Kerr S, Robinson L, *et al*. Primary biliary cirrhosis is associated with falls and significant fall related injury. *QJM* 2010;103:153–61.
9 Lai JC, Dodge JL, Sen S, *et al*. Functional decline in patients with cirrhosis awaiting liver transplantation: results from the functional assessment in liver transplantation (FrAILT) study. *Hepatology* 2016;63:574–80.
10 Zenith L, Meena N, Ramadi A, *et al*. Eight weeks of exercise training increases aerobic capacity and muscle mass and reduces fatigue in patients with cirrhosis. *Clin Gastroenterol Hepatol* 2014;12:1920–6.
11 Gerber LH, Weinstein AA, Mehta R, *et al*. Importance of fatigue and its measurement in chronic liver disease. *World J Gastroenterol* 2019;25:3669–83.
12 Dennett AM, Peiris CL, Shields N, *et al*. Moderate-intensity exercise reduces fatigue and improves mobility in cancer survivors: a systematic review and meta-regression. *J Physiother* 2016;62:68–82.
13 Razazian N, Kazeminia M, Moayedi H, *et al*. The impact of physical exercise on the fatigue symptoms in patients with multiple sclerosis: a systematic review and meta-analysis. *BMC Neurol* 2020;20:93.
14 Tomlinson D, Diorio C, Beyene J, *et al*. Effect of exercise on cancer-related fatigue: a meta-analysis. *Am J Phys Med Rehabil* 2014;93:675–86.
15 Williams FR, Vallance A, Faulkner T, *et al*. Home-based exercise therapy in patients awaiting liver transplantation: protocol for an observational feasibility trial. *BMJ Open* 2018;8:e019298.
16 Jacoby A, Rannard A, Buck D, *et al*. Development, validation, and evaluation of the PBC-40, a disease specific health related quality of life measure for primary biliary cirrhosis. *Gut* 2005;54:1622–9.
17 Mells GF, Pells G, Newton JL, *et al*. Impact of primary biliary cirrhosis on perceived quality of life: the UK-PBC national study. *Hepatology* 2013;58:273–83.
18 Hirschfield GM, Dyson JK, Alexander GJM, *et al*. The British Society of Gastroenterology/UK-PBC primary biliary cholangitis treatment and management guidelines. *Gut* 2018;67:1568–94.
19 Fisk JD, Doble SE. Construction and validation of a fatigue impact scale for daily administration (D-FIS). *Qual Life Res* 2002;11:263–72.
20 Younossi ZM, Guyatt G, Kiwi M, *et al*. Development of a disease specific questionnaire to measure health related quality of life in patients with chronic liver disease. *Gut* 1999;45:295–300.
21 Snaith RP. The hospital anxiety and depression scale. *Health Qual Life Outcomes* 2003;1:29.
22 Johns MW. A new method for measuring daytime sleepiness: the Epworth Sleepiness scale. *Sleep* 1991;14:540–5.
23 Singh SJ, Morgan MD, Scott S, *et al*. Development of a shuttle walking test of disability in patients with chronic airways obstruction. *Thorax* 1992;47:1019–24.
24 Hanson LC, Taylor NF, McBurney H. The 10m incremental shuttle walk test is a highly reliable field exercise test for patients referred to cardiac rehabilitation: a retest reliability study. *Physiotherapy* 2016;102:243–8.
25 Ronai P, Gallo PM. The short physical performance battery (assessment). *ACSMs Health Fit J* 2019;23:52–6.
26 Hlatky MA, Boineau RE, Higginbotham MB, *et al*. A brief self-administered questionnaire to determine functional capacity (the Duke activity status index). *Am J Cardiol* 1989;64:651–4.
27 Scherr J, Wolfarth B, Christle JW, *et al*. Associations between Borg's rating of perceived exertion and physiological measures of exercise intensity. *Eur J Appl Physiol* 2013;113:147–55.

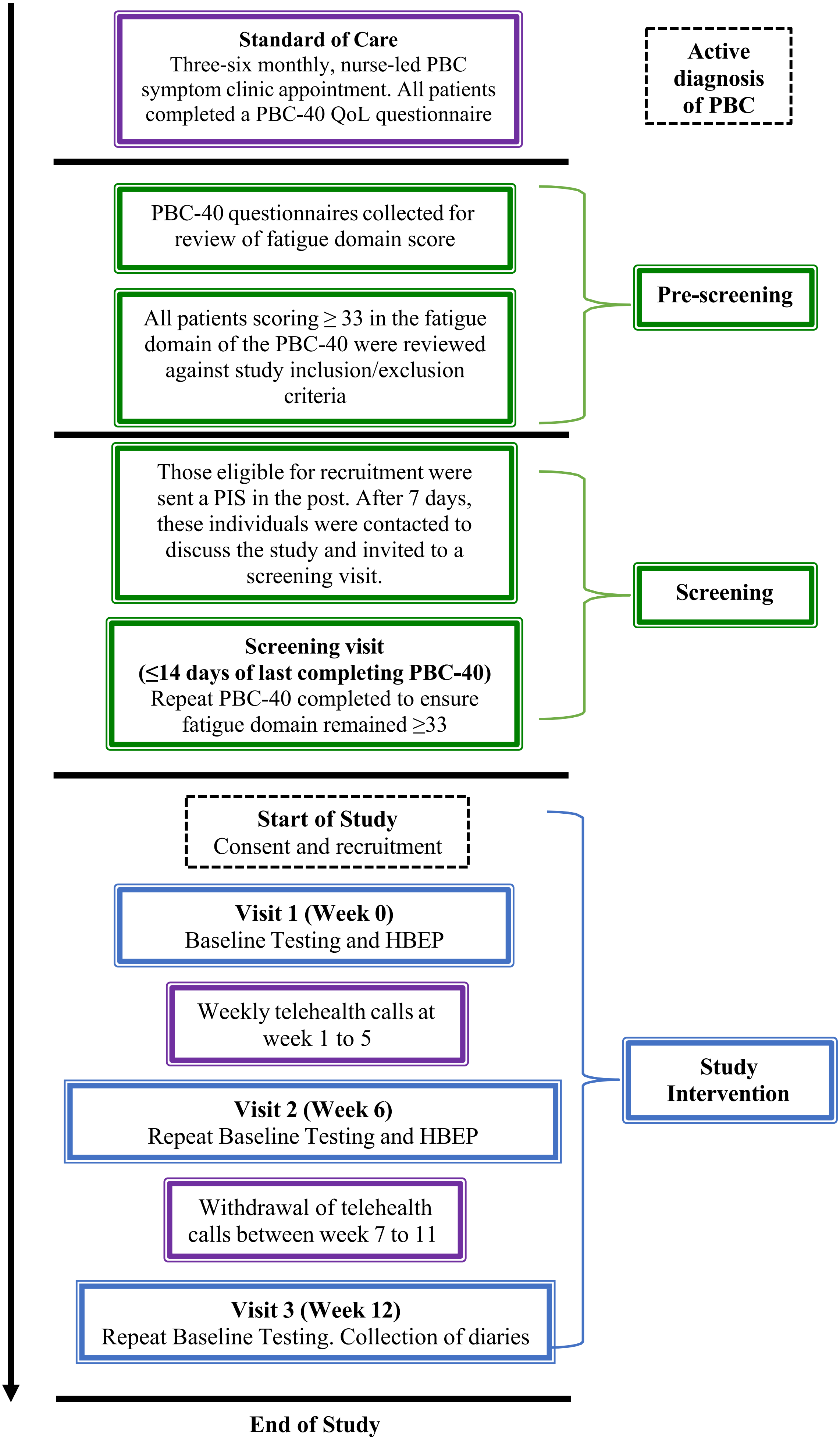

Fig. S1: Participant identification, recruitment strategy and trial design. Schematic presentation of the participant timeline from pre-screening, screening, consent, and delivery and completion of trial intervention.

**6**	**No Exertion at all**
**7**	**Extremely Light**
**8**	
**9**	**Very Light**
**10**	
**11**	**Light**
**12**	
**13**	**Somewhat Hard**
**14**	
**15**	**Hard (Heavy)**
**16**	
**17**	**Very Hard**
**18**	
**19**	**Extremely Hard**
**20**	**Maximal Exertion**

Fig. S2: The Borg Rate of Perceived Exertion.
Colour-coded training zones, white a prompt to increase intensity, green being optimal (RPE 12-14), amber a prompt to reduce intensity, and red to highlight participant work effort was too high.
Abbreviations: RPE, rate of perceived exertion

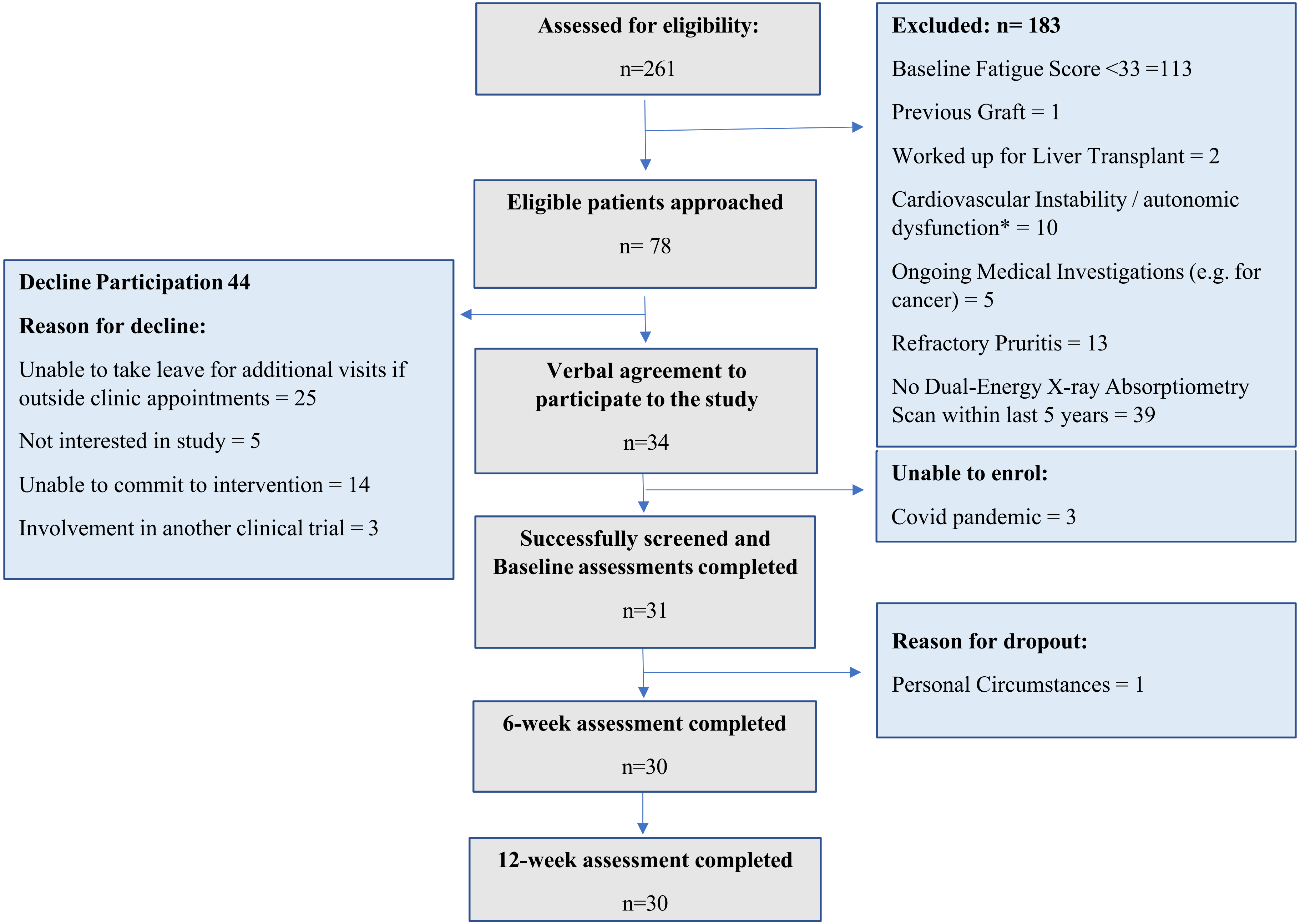

Fig. S3: Study Eligibility, Recruitment, and Retention
Schematic illustrating study course from pre-screening, screening, and completion of trial intervention.
* Individuals with evidence of other, non-PBC factors that may be contributing to fatigue were excluded; including (but not limited) to hypovitaminosis, thyroid dysfunction, untreated anaemia of any aetiology, untreated or persistent pruritus, and/or autonomic dysfunction (as evidenced by any of the following: documentary evidence in medical records, compatible symptom history with an abnormal tilt-table test, and/or a high index of suspicion raised by the treating physician).

Table S1:
Baseline characteristics of eligible patients who were recruited but declined trial participation

Participant Characteristics	Eligible and participated	Eligible but declined participation	P value *
Female sex; n (%)	29 (97%)	41 (93%)	P=.34
Age at trial entry, years	52 (44-60)	50 (41-58)	P=.17
Age at PBC diagnosis, years	46 (41-49)	47 (42-50)	P=.22
White race, n (%)	28 (93%)	40 (91%)	P=.89
Weight, kg	73.1 (57.9-88.4)	76.05 (64.4-84.7)	P=.57
BMI, kg/m²	27.6 (22.7-32.5)	29.32 (24.4-33.5)	P=.87
MELD Score	6 (6-8)	6 (6-8)	P=1.00
ALT, IU/L (ULN* 55)	33 (24-67)	35 (24-71)	P=.43
AST, IU/L (ULN 32)	41 (27-66)	43 (25-69)	P=.78
ALB, g/L (ULN 50)	42 (38-43)	40 (38-45)	P=.87
ALP IU/L (ULN 130)	153 (125-218)	171 (125.5-284.75)	P=.33
<1.9ALPxULN, n (%)	24 (80%)	37 (84%)	P=.56
≥1.9ALPxULN, n (%)	6 (20%)	7 (16%)	P=.21
Bilirubin, μmol/L (ULN 21)	12 (8-19)	10 (6.3-14.8)	P=.78
Gamma GT, IU/L (ULN 30)	105 (48-185)	100 (46-182)	P=.31
Vitamin D, ng/mL (ULN 20)	57.2 (40.2-74.2)	56.0 (39.7-70.1)	P=.35
TSH, mIU/mL (ULN 4.1)	1.34 (0.76-2.13)	1.30 (-.72-2.01)	P=.46
Creatinine, μmol/L (ULN 104)	68 (58-75)	62 (56-74)	P=.74
Haemoglobin, g/dl (ULN 16)	13.5 (12.4-14.2)	12.9 (12.4-14.6)	P=.83
AMA Positive, n (%)	22 (73%)	34 (77%)	P=.61
ANA Positive, n (%)	4 (13%)	7 (15%)	P=.52
UDCA treated, n (%)	25 (81%)	37 (84%)	P=.31
UDCA responders, n (%)	24 (80%)	37 (84%)	P=.47
UDCA intolerant, n (%)	5 (16%)	8 (18%)	P=.58
PBC 40 fatigue Score	41 (37-43)	40 (34-44)	P=.41
Globe Score	-0.73 (-1.9-0.77)	-0.73 (-1.9-0.77)	P=.89
UK PBC Risk Score 5 years (%)	1.39 (0.01-3.49)	1.41 (0.02-2.87)	P=.92
UK PBC Risk Score 10 years (%)	4.56 (0.04-11.22)	4.67 (0.06-12.89)	P=.86
UK PBC Risk Score 15 years (%)	8.32 (0.08-19.87)	8.39 (0.10-21.24)	P=.71

* Fisher's exact test were used to analyse differences between categorical variables, and the Mann-Whitney U test for analysing differences two groups of unpaired continuous variables.

ALT, alanine transaminase; AMA, anti-mitochondrial antibody; ANA, anti0nuclear antibody; AST, aspartate aminotransferase; BMI, body mass index; gamma GT, gamma glutamyltransferase; LLN, lower limit of normal; MELD, model for end-stage liver disease; PBC; primary biliary cholangitis; TSH, thyroid stimulating hormone UDCA, ursodeoxycholic acid; ULN, upper limit of normal.

Table S2:
Regression analysis of baseline covariates associated with a reduction in fatigue severity

Baseline covariate	Odds Ratio	95% CI		P value
Female sex *	2.5e8	0.00	- *	P=1.00
Age at trial entry	0.91	0.78	1.08	P=.32
Age at diagnosis	1.03	0.91	1.15	P=.67
Weight, kg	1.05	0.95	1.15	P=.32
BMI, kg/m^2	1.06	0..82	1.36	P=.68
MELD Score	1.9e9	0.00	-**	P=1.00
ALT	1.02	0.98	1.06	P=.41
AST	1.02	0.97	1.07	P=.42
Albumin	1.02	0.92	1.12	P=.78
ALP	1.00	0.99	1.00	P=.22
Bilirubin	1.32	0.93	1.88	P=.19
Gamma GT	1.00	0.99	1.01	P=.64
Vitamin D	1.00	0.97	1.03	P=.92
TSH	1.71	0.51	5.79	P=.39
Creatinine	1.02	0.92	1.13	P=.75
Haemoglobin	1.05	0.96	1.15	P=.30
UDCA treated	1.47	0.78	3.87	P=.43
UDCA response met (Paris-II)	1.01	0.46	2.76	P=.22
ALP ≥1.9 ULN	0.58	0.06	8.40	P=.79
PBC Globe Score	1.44	0.57	3.65	P=.44
UK PBC Risk Score 5 years	1.81	0.56	5.85	P=.32
UK PBC Risk Score 10 years	1.20	0.84	1.71	P=.31
UK PBC Risk Score 15 years	1.11	0.91	1.35	P=.30
PBC 40 Baseline Fatigue Score	0.94	0.79	1.13	P=.52
ESS	1.02	0.84	1,23	P=.88
ISWT	1.00	1.00	1.01	P=.41
DASI	0.67	0.38	1.18	P=.17
HADS	0.99	0.86	1.14	P=.84
SPPB	1.09	0.41	2.80	P=.89
Chair Stands	1.42	0.44	4.61	P=.56

Table reflects the results of logistic regression analysis performed on baseline covariates, and the odds of patients meeting the primary efficacy outcome measure of a PBC-40 fatigue domain score reduction of ≥5 points at week 12.

* Unable to determine the upper boundary of the CI due to only one male participant.
** Unable to determine the upper boundary of the CI due to MELD scores being too similar between participants (all but one having a baseline MELD score of 6).
ALT, alanine transaminase; AMA, anti-mitochondrial antibody; ANA, anti0nuclear antibody; AST, aspartate aminotransferase; BMI, body mass index; DASI Duke Activity Status Index; ESS, Epworth Sleepiness Score; gamma GT, gamma glutamyltransferase; HADS, Hospital Anxiety and Depression Score; ISWT, Incremental Shuttle Walking Test; LLN, lower limit of normal; MELD, model for end-stage liver disease; PBC, primary biliary cholangitis; SPPBT, Short Physical Performance Battery; TSH, thyroid stimulating hormone; UDCA, ursodeoxycholic acid; ULN, upper limit of normal.

Ymchwil Iechyd a Gofal Cymru
Health and Care Research Wales

NHS
Health Research Authority

Miss Alice Elizabeth Vallance
Senior Physiotherapist- Team Lead Specialist Surgery
Univeristy Hospital Birmingham
Mindelsohn Way
Edgbaston
B15 2WB

Email: hra.approval@nhs.net
Research-permissions@wales.nhs.uk

02 January 2019

Dear Miss Vallance

HRA and Health and Care Research Wales (HCRW) Approval Letter

**Study title:**	**EXerCise Intervention in cholesTatic LivEr Disease: The EXCITED study**
**IRAS project ID:**	**253115**
**REC reference:**	**18/LO/2109**
**Sponsor**	**University Hospitals Birmingham**

I am pleased to confirm that **HRA and Health and Care Research Wales (HCRW) Approval** has been given for the above referenced study, on the basis described in the application form, protocol, supporting documentation and any clarifications received. You should not expect to receive anything further relating to this application.

How should I continue to work with participating NHS organisations in England and Wales?
You should now provide a copy of this letter to all participating NHS organisations in England and Wales, as well as any documentation that has been updated as a result of the assessment.

This is a single site study sponsored by the site. The sponsor R&D office will confirm to you when the study can start following issue of HRA and HCRW Approval.

It is important that you involve both the research management function (e.g. R&D office) supporting each organisation and the local research team (where there is one) in setting up your study. Contact details of the research management function for each organisation can be accessed here.

How should I work with participating NHS/HSC organisations in Northern Ireland and Scotland?
HRA and HCRW Approval does not apply to NHS/HSC organisations within the devolved administrations of Northern Ireland and Scotland.

If you indicated in your IRAS form that you do have participating organisations in either of these devolved administrations, the final document set and the study wide governance report (including this letter) has been sent to the coordinating centre of each participating nation. You should work with the relevant national coordinating functions to ensure any nation specific checks are complete, and with each site so that they are able to give management permission for the study to begin.

Please see IRAS Help for information on working with NHS/HSC organisations in Northern Ireland and Scotland.

How should I work with participating non-NHS organisations?
HRA and HCRW Approval does not apply to non-NHS organisations. You should work with your non-NHS organisations to obtain local agreement in accordance with their procedures.

What are my notification responsibilities during the study?
The document "*After Ethical Review – guidance for sponsors and investigators*", issued with your REC favourable opinion, gives detailed guidance on reporting expectations for studies, including:

- Registration of research
- Notifying amendments
- Notifying the end of the study

The HRA website also provides guidance on these topics, and is updated in the light of changes in reporting expectations or procedures.

I am a participating NHS organisation in England or Wales. What should I do once I receive this letter?
You should work with the applicant and sponsor to complete any outstanding arrangements so you are able to confirm capacity and capability in line with the information provided in this letter.

The sponsor contact for this application is as follows:

Name: Dr Matthew Armstrong
Tel: 07968470622
Email: matthew.armstrong@uhb.nhs.uk

Who should I contact for further information?
Please do not hesitate to contact me for assistance with this application. My contact details are below.

Your IRAS project ID is **253115**. Please quote this on all correspondence.

Yours sincerely

Kevin Ahmed
Assessor

Telephone: 0207 104 8171
Email: hra.approval@nhs.net

Copy to: *Dr Matthew Armstrong, Sponsor Contact, University Hospital Birmingham*
Dr Chris Counsell, R&D Contact, University Hospital Birmingham

List of Documents

The final document set assessed and approved by HRA and HCRW Approval is listed below.

Document	Version	Date
GP/consultant information sheets or letters [GP Letter]	2.0	15 December 2018
IRAS Application Form [IRAS_Form_20122018]		20 December 2018
Letter from funder [Intercept Offer]	1.0	19 August 2018
Letter from funder [EXCITED grant agreement]		
Letters of invitation to participant [Letter of Invitation]	2.0	17 December 2018
Other [PI CV]	1.0	10 October 2018
Other [ISWT Proforma]	1.0	10 October 2018
Other [Telephone Questionnaire and Transcript]	1.0	10 October 2018
Other [Clinical Supervisor CV]	1.0	15 December 2018
Other [Cover Letter]	1.0	17 December 2018
Participant consent form [Consent Form]	2.0	15 December 2018
Participant information sheet (PIS) [Participant Information Sheet]	2.0	15 December 2018
Research protocol or project proposal [Protocol]	1.0	10 October 2018
Summary CV for Chief Investigator (CI) [CI CV]	1.0	10 October 2018
Summary, synopsis or diagram (flowchart) of protocol in non technical language [Flow Chart of Plan]	1.0	10 October 2018
Validated questionnaire [PBC 40 Questionnaire]	1.0	10 October 2018
Validated questionnaire [HADS]	1.0	10 October 2018
Validated questionnaire [DASI]	1.0	10 October 2018
Validated questionnaire [Epworth Sleep Scale]	1.0	10 October 2018
Validated questionnaire [Fatigue IMpact Scale]	1.0	10 October 2018
Validated questionnaire [Chronic Disease Questionnaire]	1.0	10 October 2018

Summary of assessment

The following information provides assurance to you, the sponsor and the NHS in England and Wales that the study, as assessed for HRA and HCRW Approval, is compliant with relevant standards. It also provides information and clarification, where appropriate, to participating NHS organisations in England and Wales to assist in assessing, arranging and confirming capacity and capability.

Assessment criteria

Section	Assessment Criteria	Compliant with Standards	Comments
1.1	IRAS application completed correctly	Yes	No comments
2.1	Participant information/consent documents and consent process	Yes	No comments
3.1	Protocol assessment	Yes	No comments
4.1	Allocation of responsibilities and rights are agreed and documented	Yes	This is a non-commercial single site study taking place in the NHS where that single NHS organisation is also the study sponsor. Therefore no study agreements are required.
4.2	Insurance/indemnity arrangements assessed	Yes	No comments
4.3	Financial arrangements assessed	Yes	External study funding has been secured from Intercept Pharmaceuticals.
5.1	Compliance with the Data Protection Act and data security issues assessed	Yes	No comments
5.2	CTIMPS – Arrangements for compliance with the Clinical Trials Regulations assessed	Not Applicable	No comments
5.3	Compliance with any applicable laws or regulations	Yes	No comments

Section	Assessment Criteria	Compliant with Standards	Comments
6.1	NHS Research Ethics Committee favourable opinion received for applicable studies	Yes	No comments
6.2	CTIMPS – Clinical Trials Authorisation (CTA) letter received	Not Applicable	No comments
6.3	Devices – MHRA notice of no objection received	Not Applicable	No comments
6.4	Other regulatory approvals and authorisations received	Not Applicable	No comments

Participating NHS Organisations in England and Wales

This provides detail on the types of participating NHS organisations in the study and a statement as to whether the activities at all organisations are the same or different.
If this study is subsequently extended to other NHS organisation(s) in England or Wales, an amendment should be submitted, with a Statement of Activities and Schedule of Events for the newly participating NHS organisation(s) in England or Wales.<br><br>The Chief Investigator or sponsor should share relevant study documents with participating NHS organisations in England and Wales in order to put arrangements in place to deliver the study. The documents should be sent to both the local study team, where applicable, and the office providing the research management function at the participating organisation.<br><br>If chief investigators, sponsors or principal investigators are asked to complete site level forms for participating NHS organisations in England and Wales which are not provided in IRAS, the HRA or HCRW websites, the chief investigator, sponsor or principal investigator should notify the HRA immediately at hra.approval@nhs.net or HCRW at Research-permissions@wales.nhs.uk. We will work with these organisations to achieve a consistent approach to information provision.

Principal Investigator Suitability

This confirms whether the sponsor position on whether a PI, LC or neither should be in place is correct for each type of participating NHS organisation in England and the minimum expectations for education, training and experience that PIs should meet (where applicable).
A Principal Investigator should be appointed at study sites<br><br>GCP training is <u>not</u> a generic training expectation, in line with the HRA statement on training expectations.

HR Good Practice Resource Pack Expectations

This confirms the HR Good Practice Resource Pack expectations for the study and the pre-engagement checks that should and should not be undertaken
As a non-commercial undertaken by local staff, it is unlikely that letters of access or honorary research contracts will be applicable, except where local network staff employed by another Trust (or University) are involved (and then it is likely that arrangements are already in place). Where arrangements are not already in place, network staff (or similar) undertaking any of the research activities listed in A18 or A19 of the IRAS form be expected to obtain a Letter of Access based on standard DBS checks and occupational health clearance would be appropriate.

Other Information to Aid Study Set-up

This details any other information that may be helpful to sponsors and participating NHS organisations in England to aid study set-up.
The applicant has indicated that they do not intend to apply for inclusion on the NIHR CRN Portfolio.

(RPAv46)

l-..j
Dr M Armstrong
Hepatology - Consultant Transplant Physician
Room 39, 3rd Floor.
Nuffield House
Queen Elizabeth Hospital
University Hospitals Birmingham NHS Foundation Trust
Edgbaston
Birmingham

UHB Research Governance Office
1st Floor, Institute of Translational Medicine
Heritage Building
Queen Elizabeth Hospital Birmingham
Mindelsohn Way
Edgbaston
Birmingham B15 2TH
Tel. 0121 371 4185

Research Project Authorisation

Project reference: RRK **6589**

Main Ethics Committee Reference	18/LO/2109
IRAS Project ID	253115

5 April 2019

Dear Dr M Armstrong

EXerCise Intervention in cholesTatic LivEr Disease: The EXCITED study

Thank you for submitting details of your proposed research project, which I am happy to authorise on behalf of University Hospitals Birmingham; this includes confirmation of Capacity and Capability under the HRA Approval process.

Approval covers the following site(s) only: **Queen Elizabeth Hospital Birmingham**

The following main document versions were reviewed (note this is not a complete list of all documents submitted):

Protocol- version: V1.0 10/10/18
Participant information sheet (main) - version: V2.0 15/12/18
Participant consent form (main) - version: V2.0 15/12/18

Acv1/18

Sponsorship

University Hospitals Birmingham NHS Trust has agreed to act as sponsor for this study. We believe this is a single site study. If you wish to include sites outside University Hospitals Birmingham then you must provide details of potential sites to the R&D Office, the main research ethics committee, and if necessary to the regulatory authorities. Note, however, that sites outside University Hospitals Birmingham must not start recruiting into the study until there has been formal authorisation from the R&D Office at University Hospitals Birmingham. We will require a copy of the approval letter from the R&D Department of the relevant Trust. If the study is a clinical trial of a medicine then we will also require a signed clinical trial agreement which we will draw up between UHB and the other Trust; for other types of study a clinical study agreement would not normally be required.

Indemnity arrangements.

RRK.6589

Researchers who hold substantive or honorary contracts with University Hospital Birmingham (UHBT) will be covered against claims of negligence by patients of UHBT under the Clinical Negligence Scheme for Trusts (CNST). This scheme does not cover 'no fault' compensation and the Trust is precluded from takirlg out separate insurance to cover this. Any patient or volunteer taking part in the study is entitled to know that if they suffered injury as a result of participating in the study they would first have to prove negligence in a court of law before they could gain compensation.

If the study involves patients 6f any other Trust or healthcare organisation, you will need to.confirm the indemnity arrangements with that organisation.

Pharmacy
If your study involves Pharmacy then you must ensure that they are ready to initiate the study before the first patients are recruited.

Medical Devices
Any medical devices used specifically for this study, whether purchased, loaned or borrowed must be registered with the Medical Engineering Department. Equipment must not be used until it has completed formal acceptance testing by Medical Engineering. A calibration and maintenance schedule must be drawn up and agreed with Medical Engineering in accordance with the manufacturer's recommendations. There should be a forma) maintenance contract in place if maintenance is to be carried out by external contractors (including the equipment manufacturers). If, at the end of the study, the equipment is transferred or disposed of, details must be sent to Medical Engineering to amend the equipment asset register.

Reporting Adverse Events
If this study involves an intervention in the treatment of patients then you must ensure that any serious adverse events, regardless of whether you believe the event is related to the research or the intervention, are reported according to the Trust's policy on reporting research-related adverse events. Please see attached memo. Note that you must also follow any SAE reporting requirements stipulated by the sponsor.

A copy of the Trust policy may be obtained from the R&D office and is also available on the R&D section of the Trust's intranet and internet sites. A copy of a blank SAE form is enclosed, this may be used if it is not possible to report the event-through the Trust's online reporting system.

Drugs and Treatment outside the study
Approval for the study to commence cannot be taken to imply approval for the same form of treatment to continue beyond the end of the study, or for patients who are not part of the study. If it is likely that continuing treatment is required at the end of the study, then it is the Principal Investigator's responsibility to ensure that study participants are fully aware of the types of treatment that would be available to them.

Research Governance
You should ensure that you and your research team abide by the Trust policies on research governance. These are available from the R&D Office and on the R&D section of the Trust's intranet and internet sites (www.uhb.nhs.uk/research)

Study Files
You must set up and maintain a study file containing the essential documents needed to facilitate a full audit of the conduct of the study. The minimum requirements for the content and layout of the study file are set out in the enclosed documents. This file may be audited at short notice by the R&D Office, the sponsor, or regulatory authorities.

Delegated Duties Log
You must maintain a list of all those people who have responsibility for delivering any study-related tasks set out in the protocol. The log must list the names of the individuals, their roles and responsibilities, the date

they started working on the study, and, if appropriate, the date they finished. Each entry must be signed by the person accepting the responsibilities. Note that anyone who is involved in the direct care of patients must hold a substantive or honorary contract with University Hospitals Birmingham.

PICS and Accrual Records

Research studies are now listed on a separate research tab on the trust's Prescribing, Information and Communication System (PICS). When a participant is consented or recruited into this study you must ensure that this is promptly recorded on PICS. If you have any queries about how to do this please contact the PICS training team (PICSTrainingTeam@uhb.nhs.uk). The consented date and recruitment date may be different if screening procedures are required after consenting to confirm eligibility for a study.

The R&D Governance Office will use anonymised records fr9m PICS to update central recruitment records on the UKCRN Portfolio. From April 2017 this will be the only way of recording recruitment on portfolio studies so it is essential that PICS records are accurate.

You should separately keep accurate records on the study file of recruitment and participation in your study. There should be a record, with dates, of patients approached, consented, screened, recruited, completed, and dropped out as appropriate.

Annual Reports

The R&D Office will request information about progress with the study in 6 months, 12 months and annually thereafter. Approval for this study may be withdrawn if you do not complete and return reports when requested.

Protocol Breaches

Serious protocol breaches must be reported to the R&D office as soon as possible and must be notified to the Chief Investigator and Sponsor immediately you become aware of them. If you are the Chief Investigator you must notify the Ethics Committee within 7 days and, for CTIMP studies, you must notify the MHRA within 7 days. A serious breach is one that is likely to affect to a significant degree the mental or physical integrity of the research participants or the scientific value of the study. A report of a serious breach should identify measures taken to correct the consequences of the breach and measures to prevent future similar breaches (a so-called 'CAPA' log). Minor protocol breaches should be recorded in your st�dy file.

Urgent Safety Measure

If necessary, appropriate urgent safety measure to protect clinical trial subjects from any immediate hazard to their health and safety can be taken immediately without waiting for Ethics Committee, Regulatory Authority or R&D approval. However you must inform the R&D Office, Chief Investigator, Sponsor, Ethics Committee and MHRA, as appropriate, in writing within 3 days.

Protocol Amendments

Trust approval will usually automatically cover minor protocol amendments but you must send details to the R&D office for information. Details of all substantial amendments must be sent to the R&D Office for authorisation together with copies of the ethics app.roval and/or regulatory approval for the amendments and any revised documentation. The R&D office will acknowledge all amendments. A substantial amendment is defined by NRES (the National Research Ethics Service) and would include any change that could affect the safety, conduct or the resource implications of the study.

Duration

It is expected that the study will begin at University Hospital Birmingham within 12 months of Trust authorisation. If there is a long delay in starting the study, the Trust may consider withdrawing authorisation for the study. Unless explicitly withdrawn, Trust approval lasts for as long as the study has valid ethics committee and regulatory approval.

End of Study

R&D Office
Head of R&D Governance: Dr Christopher Counsel!
Head of R&D Operations: Joanne Plumb
R&D Office, 1st Floor, ITM, Heritage Building, Queen Elizabeth Hospital Birmingham, Edgbaston Birmingham B15 2WG
Tel: 0121 371 4185 Fax:0121 371 4204 *Email:* R&D@uhb.nhs.uk
Website: www.research.uhb.nhs.uk
Projects database: //uhb/userdata/R & D/R&D database/distributed database 2002.mdb

RRK6589

According to information you have provided, this study is expected to end in March 2020 and the minimum recruitment target is 40. The R&D Office will request a final report shortly after this date. If the study ends for any reason before this date you must notify the R&D Office. Note that the Chieflnvestigator for the whole study is required to provide an end of study report to the main research ethics committee and regulatory authorities.

Archiving

For studies designated as a Clinical Trial of an Investigational Medicinal Product(CTIMP), it is a legal requirement to retain essential documents for at least 5 years after the declared end of the study. The sponsor or regulatory authorities may insist on a longer retention period for a particular study. For all other types of study there are no statutory requirements but generally accepted good practice guidelines recommend that documents are retained for at least 5 years. Documents must be archived in a way such that they can be readily accessed(24 hours notice) if required for audits or regulatory purposes. The costs of archiving is borne by the Principal or Lead Investigator and should be taken into acco1,mt when applying for research grants or seeking other forms of funding. For CTIMPs, there must be a named archivist, approved by the sponsor, who is responsible for setting up and controlling the archive.

Health Records Labelling

The Health Records of study subjects are retained according to the Trust's "Health Records Management Policy"; for patients in research studies the retention period is 15 years after the last treatment or consultation related to the study. The Principal Investigator must ensure that all records for patients involved in a study are clearly labelled to ensure that the retention policy can be followed.

Cover for absence

If the Principal Investigator is likely to be absent and out of contact for a prolonged period(> 2 weeks), the PI must either explicitly suspend patient recruitment and patient-related activity in the study, or explicitly delegate the responsibilities of Principal Investigator to a named deputy. The PI must be satisfied that their deputy is sufficiently qualified through education, training and experience to take on the role of PI. These periods of absence and delegation must be recorded in the study file.

Website entry

R&D Office
Head of R&D Governance: Dr Christopher Counsel!
Head of R&D Operations: Joanne Plumb
R&D Office, 1st Floor, ITM, Heritage Building, Queen Elizabeth Hospital Birmingham, Edgbaston Birmingham B15 2WG
Tel: 0121 371 4185 Fax:0121 371 *4204.Emai/:* R&D@uhb.nhs.uk
Website: www.research.uhb.nhs.uk
Projects database: //uhb/userdata/R & D/R&D database/distributed database 2002.mdb

RRK.6589

Basic details of your study will be made available on the Trust's website at http://www.research.uhb.nhs.uk/trials/RRK6589

First patient recruitment

NHS Trusts are monitored on how quickly the first patient is recruited into an approved clinical trial. You should aim to recruit the first patient as soon as possible and at least within 40 days of the date of this letter.

Guidance Tool

The Trust R&D Office has developed a Powerpoint-based tool summarising some of the regulations relevant to clinical research. This is available at \\uhb\userdata\R & D\R&D Shared Docs\Guide to Responsibilities\Guide to Investigator Responsibilities.ppsx (requires access to the Trust's network)

Yours sincerely,

Joanne Plumb
Deputy Director of Research Development & Innovation

Enclosed: Sample study file layout
Incident Reporting & Serious Adverse Event Form

Copies to: Miss Vallance
Relevant Service Departments
Division B Manager, Lynn Willetts

R&D Office
Head of R&D Governance: Dr Christopher Counsel!
Head of R&D Operations: Joanne Plumb
R&D Office, 1st Floor, ITM, Heritage Building, Queen Elizabeth Hospital Birmingham, Edgbaston Birmingham B15 2WG
Tel: 0121 371 4185 Fax:0121 371 4204 *Email:* R&D@uhb.nhs.uk
Website: www.research.uhb.nhs.uk
Projects database: //uhb/userdata/R & D/R&D database/distributed database 2002.mdb

Incident Reporting

As from October 2010 all clinical incidents occurring at University Hospitals Birmingham should be reported through the online reporting system http://uhbhome/departments/riskmanagement/onlinereporting.aspx. This system now includes a separate section for research related events. For incidents occurring away from UHB relating to patient involved in research studies at UHB, then the paper form attached should be completed and returned to the R&D Office.

Statutory Reporting Requirements

If this study is a clinical trial of a medicine or a device then there are statutory reporting requirements. The Principal Investigator must make an assessment of the seriousness of the incident, its relatedness to the study intervention or any breach of the study protocol, and the expectedness of the event (against known characteristics of the medicine or device). An incident which is serious, related and unexpected is referred to as a SUSAR and must be reported to the Chief Investigator and Sponsor as soon possible. The CI/Sponsor are responsible for reporting SUSARs to the MHRA within 15 days of becoming aware of the event (7 days in the case of events resulting in death). SUSARs must also be reported to the manufacturer of the medicine or device if they are not the sponsor of the study.

Pregnancies

For clinical trials involving medicines (CTIMPs) for which the effect of'the IMP on an unborn child is unknown, special care should be taken to avoid pregnancies during the interventional phase of the trial. Advice on appropriate contraception should be provided to potential participants before recruitment into a study. If a female participant, or the female partner of a male participant, becomes pregnant during the interventional phase of a CTIMP, then the pregnancy must be reported to the sponsor as soon as possible. In the case of CTIMPs sponsored by University Hospitals Birmingham, pregnancies must be reported immediately to the R&D Office. Pregnancies must be followed through to term to be able to check for any birth defects that could be attributable to the treatment. Birth defects are classed as serious adverse events and may be reported as a SUSAR depending on their expectedness and relatedness to the intervention.

Serious Adverse Event Form (v2)
(Research drugs, devices and interventions)
University Hospital Birmingham NHS Trust

This form must be completed in the event of a Serious Adverse Event / Incident.
This can be defined as an untoward medical occurrence in a patient during clinical research involving a pharmaceutical product or clinical intervention that: is fatal; is life threatening; results in persistent or significant disability/ incapacity ; requires inpatient hospitalisation or prolongs a current hospitalisation; is a congenital anomaly in offspring; or an event that may jeopardise the patient or may require intervention to prevent one of the outcomes listed above.

(R&D SAE id:______)

Study Title or Trust RRK Number:

Section A - Details of Subject Affected by Serious Adverse Event

Has the Principal Investigator been informed of this event prior to completion of this form? Yes D **No**

Subject Initials:________ Risk Form Number:__________ Subject Number:_________

Section B - Details of the Serious Adverse Event

Date of Onset:________________ **Time:** ____________________________

Hospital:__________________________ **Exact Location:** ______________

Definition of Serious Adverse Event: (tick the appropriate category for the event)

**Death**	D	**Resulted in Disability**	☐	**Congenital abnormality**	D
**Life threatening**	D	**Required Hospitalisation**		**none of above**	D

Describe Event: (A summary of signs and symptoms (including severity), vital signs, diagnosis, treatment of event, concurrent treatment, other relevant medical history, details of study drug/ device. Please include the time point in the study at which the event occurred.)

Number of additional pages added, if any ____

(R&D SAE Id:__________) **Risk Form Number: _ _ _ _**

Section C - Relationship To Study Involvement

1. Was the incident related to the patient's involvement in the study?

Likely D **Possible** D **Unlikely** ☐ D

2. Is the event related to a break in the study protocol? **Possible** **Unlikely** D

3. If you answered **Possible** to number 2,please give details below

4. Was the event unexpected (i.e. not consistent with available information about the drug, device or intervention)? **Expected** D **Unexpected**

5. Action Taken Regarding Participation In Study:

Temporarily Discontinued **Date:_ _ _ _ _ _**
Decision taken by:__________

Permanently Discontinued **Date:_ _ _ _ _ _**
Decision taken by:__________

Patient Continued In Study (Please Tick Box) ☐

Section D - Outcome Of Serious Adverse Event

Recovered ☐

Event Continuing ☐

Patient Died ☐

If necessary please give additional details below:

Section E - Reporter's Details

(Please Print)

Name:____________________ **Title:__________** **Post:_______________**

Department:__________________ **Contact Number:** _ _ _ _ _ _ _ _ _ _ _ _

Please attach the completed form to a Trust Incident R,port Form and complete sectl<>ns A (Details Of The Person Affected By The Incident) and D (Details Of The Person Cofi'lpletlng The Form). Forward both forms to: Trhe R&D Office, &ueen Elizabeth HQspital Birmingham, Edgbaston, Birmingham
Forward one copy of this form to the Prlnc1pal Investigator and r:etaln one copy In the Study Flie.

(SFv4)

Study Fi le

It is a requirement of Trust authorisation of a study that the Principal Investigator establishes and maintains a study file. A dedicated member of the study team is responsible for maintaining and updating the file.

Good Clinical Practice guidelines require those documents to be collected in a Study Master File which individually and collectively permit evaluation of the conduct of a study and the quality of the data produced.

Some sponsors may provide their own study file (also known as an Investigator File, Site File, or Trial Master File) for specific studies. If it is a multicentre study, the sponsor may organise the documents into a central Tr.ial Master File and separate Site Files.

Clinical Trials of Investigational Medicinal Products {CTIMPs) fall within the remit of EU Directive 2005/28/EC (the "GCP Directive") which contains detailed requirements for filing and archiving essential documents. For CTIMPs it is a **legal** requirement to follow the requirements of the GCP Directive. The Trust recommends that as a principle of good research practice, study files for all types of studies are maintained to the standards set out in the GCP Directive.

- The site study file must be kept secure at all times.
- The local Principal Investigator must take care to ensure that only personnel authorised by them can amend the file.
- The file should be available for reference by members of the local study team as needed.
- The file must be available for inspection, with 24 hours notice, by representatives of the Sponsor, the Trust, or regulatory authority.
- The file must be retained after the end of the study (final data collection from the final research subject) for at least 5 years.

Documentation

The following good documentation principles should be followed:

- All documents in the file must be complete, legible, accurate and unambiguous
- Documents should be signed and dated as appropriate (e.g. protocol, letters, records of actions etc.)
- If stored on electronic, optical, magnetic format then suitable controls must be in place to ensure the documents cannot be altered without appropriate authorisation
- There must be an audit trail of modifications to the files
- Important documents must carry version numbers or dates (in particular the protocol, investigator brochure, subject information leaflets, subject consent forms, template case report form/data collection form)
- Version numbers must tally with those approved by regulatory authorities, including the ethics committee
- The current and previous versions of documents should be retained on file
- Previous versions should be clearly marked as no longer current together with the date they were superseded.
- Details of amendments made over the course of the study should be recorded in the appropriate section of the file. If necessary, full copies of previous versions may be retained in a separate file.

R&D Office
Head of R&D Governance: Dr Christopher Counsel!
Head of R&D Operations: Joanne Plumb
R&D Office, 1st Floor, ITM, Heritage Building, Queen Elizabeth Hospital Birmingham, Edgbaston Birmingham B15 2WG
Tel: 0121 371 4185 Fax:0121 371 4204 *Email:* R&D@uhb.nhs.uk
Website: www.research.uhb.nhs.uk
Projects database: //uhb/userdata/R & D/R&D database/distributed database 2002.mdb

There must be sufficient information on file for auditors to be able to recreate the document versions used at each time-point for the study.

- A monitor from the sponsor will usually review the accuracy and completeness of the investigator file before formally closing the study

Storage

- The file must be stored so that records remain legible and can be readily retrieved
- The stprage facilities must be secure with appropriate environmental controls and adequate protection from physical damage
- Any change in the location of the documents must be recorded to enable complete tracking

Archiving

- Named individuals approved by the sponsor are responsible for archiving
- Access to the archives must be restricted to the named responsible individuals
- The named individuals must maintain a log of the documents retained in the archive and to track movement of documents into and out of the archive
- The study file can be transferred on to alternc1te media for archiving but the transfer must be certified for accuracy and completeness
- The sponsor can determine how long the study files and all study data should be retained but it must be for a minimum of 5 years after the last clinical intervention on the last participant in the study
- Documents must not be destroyed until agreed with the sponsor

R&D Office
Head of R&D Governance: Dr Christopher Counsell
Head of R&D Operations: Joanne Plumb
R&D Office, 1st Floor, ITM, Heritage Building, Queen Elizabeth Hospital Birmingham, Edgbaston Birmingham B15 2WG
Tel: 0121 371 4185 Fax:0121 371 4204 *Email:* R&D@uhb.nhs.uk
Website: www.research.uhb.nhs.uk
Projects database: //uhb/userdata/R & D/R&D database/distributed database 2002.mdb

Abbreviations

ARSAC	Administration of Radioactive Substances Advisory Committee
CAG	NIHS. Confidentiality Advisory Group
CI	Chief Investigator
CRF	Case Record Form
CRO	Contract Research Organisation
CTA	Clinical Trial Agreement or Clinical Trial Authorisation depending on context
CTIMP	Clinical Trial of an Investigational Medicinal Product
GCP	Good Clinical Practice
GMSC	Genetics Modification Safety Committee
GTAC	Gene Therapy Advisory Committee
HFEA	Human Fertilisation and Embryology Authority
HRA	Health Research Authority
IMP	Investigational Medicinal Product
IRMER	Ionising Radiation (Medical Exposure) Regulations
MHRA	Medicines and Healthcare Products Regulatory Authority
NRES	National Research Ethics Service
PI	Principal Investigator
REC	Research Ethics Committee
SAE	Serious Adverse Event
SAR	Serious Adverse Reaction
SUSAR	Suspected Unexpected Serious Adverse Reaction

R&D Office
Head of R&D Governance: Dr Christopher Counsel!
Head of R&D Operations: Joanne Plumb
R&D Office, 1st Floor, ITM, Heritage Building, Queen Elizabeth Hospital Birmingham, Edgbaston Birmingham B15 2WG
Tel: 0121 371 4185 Fax:0121 371 4204 *Email:* R&D@uhb.nhs.uk
Website: www.research.uhb.nhs.uk
Projects database: //uhb/userdata/R & D/R&D database/distributed database 2002.mdb

Suggested Contents and Layout

Inteventional Study

This layout is applicable for any study that involves an intervention in the care of patients.
(Not all documents may be appropriate for all studies, but where the documents do exist they should be included in this file)

**1.** R&D Specific	<:;it. rilo	**Sponsor Master File**
**1.1.** Documents		
1.1.1. PI Agreement	•	
1.1.2. Clinical Director's agreement	•	
1.1.3. UHB Sponsor letter	•	
1.1.4. IRMER Schedule 8 Form		
1.1.5. ARSAC Certificate	•	
1.1.6. GMSC Risk Report (for gene theraov studies)	•	
1.1.7. Trust approval letter	o	•
1.1.8. Other R&D documents (Data Transfer Form, Treatment Continuation Form etc.(	,p	
1.1.9. R&D correspondence	•	
**2.** GCP Study File		
**2.1. Basic documents**		
**2.1.1.** Investigator Brochure*	•	•
**2.1.2.** Protocol (signed by Chief/Principal Investigator)	•	•
**2.1.3.** IRAS Application (all sections)	•	•
**2.1.4.** Participant Information Sheets & Consent Forms	•	•
**2.1.5.** Advertisement for subject recruitment	•	
**2.1.6.** Sample letters (e.g. to GP)		•
**2.1.7.** Randomisation procedures	•	•
**2.1.8.** Template Case Report Form or other data collection proformas (if not in protocol)	•	•
**2.1.9.** Normal ranges for lab tests	•	
**2.1.10.** Medical/laboratory/technical tests and procedures	•	•
**2.2. Approvals**		
2.2.1. Ethics committee approval	•	•
**2.2.2.** Health Research Authority (HRA) approval	•	•
**2.2.3.** Regulatory (MHRA) authorisation!		
**2.2.4.** Funding approval	•	•
**2.2.5.** Peer-review reports	o	
**2.2.6.** Other regulatory authority authorisations (GTAC, CAG, HFEA etc.)	o	•

·or Summary of Product Characteristics (SmPC) if the medicinal p.roduct is already licensed

R&D Office
Head of R&D Governance: Dr Christopher Counsel!
Head of R&D Operations: Joanne Plumb
R&D Office, 1st Floor, ITM, Heritage Bull.ding, Queen Elizabeth Hospital Birmingham, Edgbaston Birmingham B15 2WG
Te/; 0121 371 4185 Fax:0121 371 4204 *Email:* R&D@uhb.nhs.uk
Website: www.research.uhb.nhs.uk
Projects database: //uhb/userdata/R & D/R&D database/distributed database 2002.mdb

2.3. Studv Personnel		
2.3.1. CVs	o	•
2.3.2. Delegated duties log & Signature sheet	•	•
2.4. Agreements		•
2.4.1. Financial agreement	•	•
2.4.2. Insurance statement	9	•
2.4.3. Sponsorship statement	•	
2.4.4. Sponsor-Site agreemenU Clinical Trial Agreement	•	•
2.5. Pharmacy[1]		
2.5.1. Investigational Medicinal Product(s} characteristics	•	•
2.5.2. Instructions for handling investigational product	•	•
2.5.3. Sample of labels attached to medicinal products		•
2.5.4. Investigational products shipping and distribution records	•	•
2.5.5. Investigational products accountability record (destruction, return etc.)	o	•
2.5.6. Decoding procedures for blinded trials	•	•
2.6. Amendments		
2.6.1. Updates of Investigator Brochure	o	•
2.6.2. List of protocol amendments	9	•
2.6.3. Approvals for substantial amendments (ethics, regulatory, R&D)	D	• •
2.6.4. Updates to laboratory normal values/ranges	c	
2.6.5. Updates to medical/laboratory/technical tests and procedures	•	•
2.7. Correspondence		
2.8. Subject enrollment		
2.8.1. Subject screening log	•	
2.8.2. Subject recruitment log.	•	
2.8.3. Subject identification code	o	
2.8.4. Signed consent forms (these may be kept separately provided their location is clearly indicated in the master study file)	•	
2.8.5. Completed CRFs or location of source data	o	
2.8.6. Record of retained human tissue & fluid samples	•	
2.9. Adverse events	•	•
2.9.1. Sample SAE form		
2.9.2. SAE/SUSAR reporting procedures	•	•
2.9.3. List of Expected Serious Adverse	•	

[1] Pharmacy documents may be kept in a separate file held in pharmacy: this should be indicated in the main study file.

R&D Office
Head of R&D Governance: Dr Christopher Counsel!
Head of R&D Operations: Joanne Plumb
R&D Office, 1st Floor, ITM, Heritage Building, Queen Elizabeth Hospital Birmingham, Edgbaston Birmingham B15 2WG
Tel: **0121 371 4185 Fax:0121 371 4204** *Email:* **R&D@uhb.nhs.uk**
Website: **www.research.uhb.nhs.uk**
Projects database: **//uhb/userdata/R & D/R&D database/distributed database 2002.mdb**

	Reactions		
**2.9.4.**	Completed SAE forms	•	•
**2.9.5.**	Notification by Sponsor/Investigator to requlatorv authorities of SUSARs	•	
**2.9.6.**	Notifications by sponsor to investigators of safetv information	o	
**2.10.**	**Monitoring**		
2.10.1.	Study initiation report	•	•
**2.10.2.**	Monitoring/audit log	•	
**2.10.3.**	Audit reports	•	
**2.11.**	**Reports**		
2.11.1.	Annual reports (to ethics committee, MHRA, R&D ...)	o	
	Final report	[illegible]	•
**2.11.3.**	Publications list	o	

R&D Office
Head of R&D Governance: Dr Christopher Counsel!
Head of R&D Operations: Joanne Plumb
R&D Office, 1st Floor, ITM, Heritage Building, Queen Elizabeth Hospital Birmingham, Edgbaston Birmingham 815 2WG
Tel: 0121 371 4185 Fax:0121 371 4204 *Email:* R&D@uhb.nhs.uk
Website: www.research.uhb.nhs.uk
Projects database: //uhb/userdata/R & D/R&D database/distributed database 2002.mdb

Non-Interventlonal Study

This layout is applicable for any study that does not involve a direct intervention in the care of patients. Including, but not limited to, studies simply involving:

- questionnaires to patients or staff
- collection of human tissue
- analysis of existing data from patient records

Not all documents may be appropriate for all studies, but where the documents do exist they should be included in this file.
*Star inc;licates a document that must be on file as an absolute minimum.

1 R&D Specific
 1.1 Documents
  1.1.1 PI Agreement*
  1.1.2 Clinical Director's agreement*
  1.1.3 UHB Sponsor letter
  1.1.4 Trust approval*
  1.1.5 Other R&D documents
  1.1..6 R&D correspondence

2 GCP Study File.
 2.1 Basic documents
  2.1.1 Protocol (signed by Chief/Principal Investigator)*
  2.1.2 IRAS Application
  2.1.3 Participant Information Sheets & Consent Forms*
  2.1.4 Questionnaires
  2.1.5 Advertisement for subject recruitment
  2.1.6 Sample letters (e.g. to GP)
  2.1.7 Case Report Form or other data collection proforma (if not in protocol)
 2.2 Approvals
  2.2.1 Ethics committee approvals*
  2.2.2 Health Research Authority approval*
  2.2.3 Funding approval
  2.2.4 Peer-review reports
  2.2.5 Regulatory authority authorisations (GTAC, CAG, HFEA etc.)
 2.3 Study Personnel
  2.3.1 CVs*
  2.3.2 Delegated duties log & Signature sheet
 2.4 Agreements
  2.4.1 Sponsorship statement
  2.4.2 Financial agreement
  2.4.3 Sponsor-Site agreement
 2.5 Amendments
  2.5.1 List of protocol amendments*
  2.5.2 Approvals for substantial amendments (ethics, regulatory, R&D) *
 2.6 Correspondence
 2.7 Subject enrollment
  2.7.1 Subject screening and recruitment logs
  2.7.2 Signed consent forms (these may be kept separately provided their location is clearly indicated in the master study file)*
  2.7.3 Completed CRFs or location of source data
  2.7.4 Record of retained human tissue & fluid samples
 2.8 Monitoring
  2.8.1 Monitoring/audit log
  2.8.2 Audit reports
 2.9 Reports
  2.9.1 Annual reports (to ethics committee, MHRA, R&D ...)

R&D Office
Head of R&D Governance: Dr Christopher Counsel!
Head of R&D Operations: Joanne Plumb
R&D Office, 1st Floor, ITM, Heritage Building, Queen Elizabeth Hospital Birmingham, Edgbaston Birmingham B15 2WG
Tel: 0121 371 4185 Fax:0121 371 4204 *Email:* R&D@uhb.nhs.uk
Website: www.research.uhb.nhs.uk
Projects database: //uhb/userdata/R & D/R&D database/distributed database 2002.mdb

2.9.2 Final report
2.9.3 Publications list

R&D Office
Head of R&D Governance: Dr Christopher Counsel!
Head of R&D Operations: Joanne Plumb
R&D Office, 1st Floor, ITM, Heritage Building, Queen Elizabeth Hospital Birmingham, Edgbaston Birmingham B15 2WG
Tel: 0121 371 4185 Fax:0121 371 4204 *Email:* R&D@uhb.nhs.uk
Website: www.research.uhb.nhs.uk
Projects database: //uhb/userdata/R & D/R&D database/distributed database 2002.mdb

NHS University Hospitals Birmingham NHS Foundation Trust

PARTICIPANT INFORMATION SHEET

A home-based exercise programme attenuates fatigue in primary biliary cholangitis: The EXerCise Intervention in cholesTatic LivEr Disease (EXCITED) clinical trial

IRAS Reference Number: 253115

Principal Investigator: ***Dr. Matthew Armstrong***

Consultant Hepatologist

Queen Elizabeth Hospital Birmingham

Telephone: 0121 3712000

Co-Investigator: ***Alice Vallance***

Physiotherapy Team Lead – Specialist Surgery

Queen Elizabeth Hospital Birmingham

Telephone: 0121 371 2000

Email: alice.vallance@uhb.nhs.uk

As a patient at the University Hospital Birmingham NHS Foundation Trust, and Liver Unit you are being invited to take part in a research project. This project is part of a Masters in Research. It is important that you read all this information to have a better understanding of why this research is being completed before you agree to take part.

Who is organising the study?

The study is being organised by Miss Alice Vallance (Specialist Physiotherapist) and supporting medical team. The study will be supported by the University

Hospital Birmingham NHS Foundation Trust (UHBfT), and the Research and Governance department.

Who has reviewed the study?

All research in the NHS is looked at by an independent group of people. This group is a Research Ethics Committee, and their aim is to protect your safety, rights, wellbeing and dignity.

What is the purpose of the study?

The purpose of this study is to investigate if a home-based exercise programme helps to improve your quality of life and levels of fatigue. Fatigue effects people in different ways and can interfere with every day life. Exercise and its benefits on fatigue have been proven in other diseases and therefore will be used to see if it can improve your quality of life and fatigue. You may have received advice in the past about exercise but what makes this study unique is that you will receive an accelerometer that will help monitor the level of exercise completed. In addition, the exercise advice given to you will be something you complete at home in your own environment at a time that suits you.

Why have I been chosen?

You have been invited to take part in this study because you have symptoms of fatigue caused by liver disease and are a patient of the Birmingham Liver Unit. The aim of the study is to see if exercise can improve symptoms of fatigue and quality of life.

Do I have to take part?

It is important that you understand that your participation in this research project is completely voluntary. If you decide not to take part in this research it will have no impact on the care and treatment you receive at the hospital. If you do choose to take part you will be able to keep a copy of this information sheet and a copy of the consent form to remind you of the study information.

What happens if I decide to take part?

Up to one week after being provided with this information sheet a member of the study team will contact you to see if you would like to take part. If you agree to take part, you will be invited to come and discuss the patient information sheet and sign a consent form. This initial meeting is likely to be an additional visit to the hospital, but after this and where possible any other study visits will be on the same day as your clinic appointments to minimise any inconvenience. You will be able to ask any questions about the study before you sign the consent form so you fully understand the study and what you are agreeing to. The study intervention is 12 weeks and you will be involved in three separate study visits. On the same visit that you sign your consent form some baseline tests will be completed (Visit 1). You will be asked to complete a physical assessment that looks at your ability to exercise and answer several questionnaires that look at various different topics and are explained below.

The Physical Assessment: The Incremental Shuttle Walking Test

The Incremental Shuttle Walking Test is a walking test that involves walking between two cones that are ten metres apart. You will follow a set rhythm that is indicated by a CD recording. This will steadily get faster and you will need to steadily speed up. The test will stop if you can no longer keep up with the set pace or feel you cannot complete any more of the test. Throughout the test a

breathlessness scale called the Rate of Perceived Exertion will be used to help you tell us how breathless you feel when completing the walking test.

The Questionnaires

Fatigue Impact Scale

This questionnaire is 8 questions long and lets you rate how much your symptoms of fatigue interfere with your ability to make decisions and complete different day-to-day tasks.

The Chronic Liver Disease Questionnaire

This questionnaire is 29 questions long and lets you rate how much your symptoms of fatigue interfere with your ability to make decisions and complete different day-to-day tasks. It will give us more detailed information about how your fatigue impacts you compared to the Fatigue Impact Scale.

PBC-40 Quality of Life Tool

The PBC-40 Quality of Life Tool is a questionnaire that is 40 questions long that allows you to rate your quality of life.

Hospital and Depression Scale

The Hospital Anxiety and Depression Score is a questionnaire that is 14 questions long that help us to identify if you are feeling low in mood or anxious. It is important that you understand we do not have involvement from a psychologist or councillor but would be happy to inform your GP if this support was needed.

Epworth Scale

The Epworth Scale is a questionnaire that has 8 questions that allows you to rate how sleepy you get doing day-to-day activities.

Duke Activity Status Index (DASI)

The DASI is a questionnaire that has 12 questions that ask about your ability to complete different day-to-day tasks within the house and ability to exercise/ work.

Completing all of these questionnaires is likely to take no longer than 15-20 minutes. If you need support reading the questions one of the study team will happily assist you.

Following these questionnaires and your physical assessment you will be provided with an exercise programme. This exercise programme will be individualised to your ability and you will have the opportunity to practice this under the support of the study team to make sure you have the correct technique.

The Exercise Programme:

The exercise programme will be made up of 4-6 different body weighted exercises. This means no specialist equipment will be required simply just the weight of your own body- an example of such exercise would be a squat. Depending on your ability will depend on the body-weighted exercises given. All of the exercise programmes will be issued by a Specialist Physiotherapist within Liver Disease.

We will ask you to complete each exercise for a period of time (between 20 and 40 seconds) followed by a rest period (between 20- 60 seconds). Each exercise will be completed several times and will result in a 20-25 minute exercise programme. The amount of time you exercise and rest will be determined by the Specialist Physiotherapist and will be individualised to your needs. You will be advised to complete the exercise programme 2-3 times a week.

Once you have been taught your individualised programme you will be given a paper diary and paper copy of the exercises to help support you complete this programme. In addition we will also give you an accelerometer that will monitor the amount of activity you complete.

The Accelerometer

The accelerometer is a wrist worn device that has been supplied by a company called GENEActiv. This device does not have a display screen and therefore does not give you any information about the level of activity you are completing. Whilst worn the device will be recording your levels of activity and this will be taken from the device once you have finished completing your exercise programme for 12 weeks.

To complete all aspects of Visit 1 will take approximately 90 minutes of your time. Following this, you will be contacted weekly for 6 weeks to discuss your progress and any problems you have encountered completing your programme. Each telephone call is estimated to take no longer than 20 minutes.

Visit 2

Following the initial 6-week intervention you will be invited back to recomplete both the physical assessment and questionnaires from Visit 1. The exercise programme you have been completing will be reviewed to ensure you continue to have good technique. Any questions will be answered and then you will be asked to complete the programme for a further 6 weeks without telephone support.

Visit 3

6 weeks following Visit 2 you will be invited back to complete your final physical assessment and questionnaires. The accelerometer will be handed back to the study team. This will be the end of the study.

What are the possible benefits?

We are unable to predict if the study will change your levels of fatigue but it is hoped it will reduce your symptoms. It may also give us some evidence that suggests exercise is a useful treatment option in treating fatigue.

What if something goes wrong?

We are confident that by taking part in this study there will be no negative impacts. If you become distressed or upset during any part of the study you can seek support from the principle investigator. Alternatively you can contact the Patient Advisory and Liaison Service (PALS) at the Hospital. The PALS department can be contacted through the main switchboard number on 0121 371 2000.

Confidentiality

All information relating to your participation will be kept completely confidential in line with UHBfT information governance protocols. Each participant will be assigned a unique participation number and all data will be recorded under this number. When the study is written up it will include no personal information or data that can identify you.

UHBfT is the sponsor for this study and based in England. We will be using information from you and/or your medical records in order to undertake this study and will act as the data controller for this study. This means that we are responsible for looking after your information and using it properly. UHBfT will keep identifiable information about you for 5 years after the study has finished. Your rights to access, change or move your information are limited, as we need to manage your information in specific ways in order for the research to be reliable and accurate. If you withdraw from the study, we will keep the information about

you that we have already obtained. To safeguard your rights, we will use the minimum personally-identifiable information possible.
You can find out more about how we use your information at www.research.uhb.nhs.uk/legal-information/privacy-policy.

Researchers at the Queen Elizabeth Hospital Birmingham will use your name, NHS number and contact details to contact you about the research study, and make sure that relevant information about the study is recorded for your care, and to oversee the quality of the study. Individuals from UHBfT and regulatory organisations may look at your medical and research records to check the accuracy of the research study.

<u>What if I have any questions?</u>

If you have any questions about participation in this study or concerns about the way it has been carried out, you can contact me at any time.

Miss Alice Vallance
University Hospital Birmingham NHS Foundation Trust,
Mindelson Way,
Edgbaston,
Birmingham B15 2WB. Tel: 0121 371 2000.
E-Mail: alice.vallance@uhb.nhs.uk
